# EMT and EndMT: Emerging Roles in Age-Related Macular Degeneration

**DOI:** 10.3390/ijms21124271

**Published:** 2020-06-16

**Authors:** Daisy Y. Shu, Erik Butcher, Magali Saint-Geniez

**Affiliations:** 1 Schepens Eye Research Institute of Massachusetts Eye and Ear, Boston, MA 02114, USA; Daisy_Shu@MEEI.HARVARD.EDU (D.Y.S.); Erik_Butcher@MEEI.HARVARD.EDU (E.B.); 2 Department of Ophthalmology, Harvard Medical School, Boston, MA 02114, USA; 3 Harvard John A. Paulson School of Engineering and Applied Sciences, Harvard University, Cambridge, MA 02138, USA

**Keywords:** age-related macular degeneration, epithelial–mesenchymal transition, endothelial–mesenchymal transition, subretinal fibrosis, transforming growth factor-beta

## Abstract

Epithelial–mesenchymal transition (EMT) and endothelial–mesenchymal transition (EndMT) are physiological processes required for normal embryogenesis. However, these processes can be hijacked in pathological conditions to facilitate tissue fibrosis and cancer metastasis. In the eye, EMT and EndMT play key roles in the pathogenesis of subretinal fibrosis, the end-stage of age-related macular degeneration (AMD) that leads to profound and permanent vision loss. Predominant in subretinal fibrotic lesions are matrix-producing mesenchymal cells believed to originate from the retinal pigment epithelium (RPE) and/or choroidal endothelial cells (CECs) through EMT and EndMT, respectively. Recent evidence suggests that EMT of RPE may also be implicated during the early stages of AMD. Transforming growth factor-beta (TGFβ) is a key cytokine orchestrating both EMT and EndMT. Investigations in the molecular mechanisms underpinning EMT and EndMT in AMD have implicated a myriad of contributing factors including signaling pathways, extracellular matrix remodelling, oxidative stress, inflammation, autophagy, metabolism and mitochondrial dysfunction. Questions arise as to differences in the mesenchymal cells derived from these two processes and their distinct mechanistic contributions to the pathogenesis of AMD. Detailed discussion on the AMD microenvironment highlights the synergistic interactions between RPE and CECs that may augment the EMT and EndMT processes in vivo. Understanding the differential regulatory networks of EMT and EndMT and their contributions to both the dry and wet forms of AMD can aid the development of therapeutic strategies targeting both RPE and CECs to potentially reverse the aberrant cellular transdifferentiation processes, regenerate the retina and thus restore vision.

## 1. Introduction

Subretinal fibrosis demarcates the end-stage of age-related macular degeneration (AMD), resulting in permanent vision loss [1]. Fibrosis is the product of an aberrant and excessive wound healing response characterized by the presence of motile and contractile mesenchymal cells termed myofibroblasts [2]. A dramatic remodeling of the extracellular matrix (ECM) is driven by the coordinated activity of proteolytic enzymes called matrix metalloproteases (MMPs) and tissue inhibitors of MMPs (TIMPs) [3]. This process restores the protective barrier, but can also progressively remodel and destroy normal tissue leading to contracture and distortion of tissue architecture. Since retinal visual function is achieved through highly organized anatomical layers and tightly coordinated cellular interactions, subretinal fibrosis inevitably leads to profound and often irreversible visual impairment. Currently, the only treatment for subretinal fibrosis is through invasive surgical intervention and thus, research in unravelling the molecular mechanisms underpinning aberrant retinal wound healing is imperative for the development of non-invasive drug-based therapies [1].

Predominant in subretinal fibrotic lesions are myofibroblasts, which are not normally present in adult tissues and thus, their cellular origins remain an ongoing debate. Extensive literature supports the role of epithelial–mesenchymal transition (EMT) in myofibroblast production [3]. In the context of AMD, retinal pigment epithelial (RPE) cells lose their cell–cell adhesions and apical–basal polarity, transforming into mesenchymal cells through EMT [4]. Another emerging hypothesis is the role of endothelial–mesenchymal transition (EndMT) in contributing to the mesenchymal cell population [5]. In neovascular AMD (nAMD), angiogenesis initiates inflammatory cell recruitment and increases oxygen supply and nutrients to the macular region [6]. Neovessels sprouting from either the choroid or the deep retinal vessels form choroidal neovascular membranes (CNV) or intraretinal angiomatous proliferative lesions, respectively. EndMT of choroidal endothelial cells (CECs) or retinal endothelial cells could contribute to the mesenchymal cell population in subretinal fibrotic lesions. These leaky neovessels also contribute to retinal edema, hemorrhage and further potentiate the pathological wound healing response.

Importantly, the process of EMT in RPE is not limited to subretinal fibrosis in AMD but rather, has also been extensively studied and described in the context of proliferative vitreoretinopathy (PVR) [7], epiretinal membrane (ERM) [8], and retinal fibrosis in proliferative diabetic retinopathy (PDR) [9]. Similarly, EndMT has been studied in both choroidal and retinal endothelial cells in numerous ocular angiogenic diseases in addition to nAMD, including proliferative diabetic retinopathy and retinopathy of prematurity (ROP) [5,10]. Hence, mechanistic insights in EMT and EndMT gleaned from studies outside of AMD may help to guide future AMD research.

**Figure 1:**
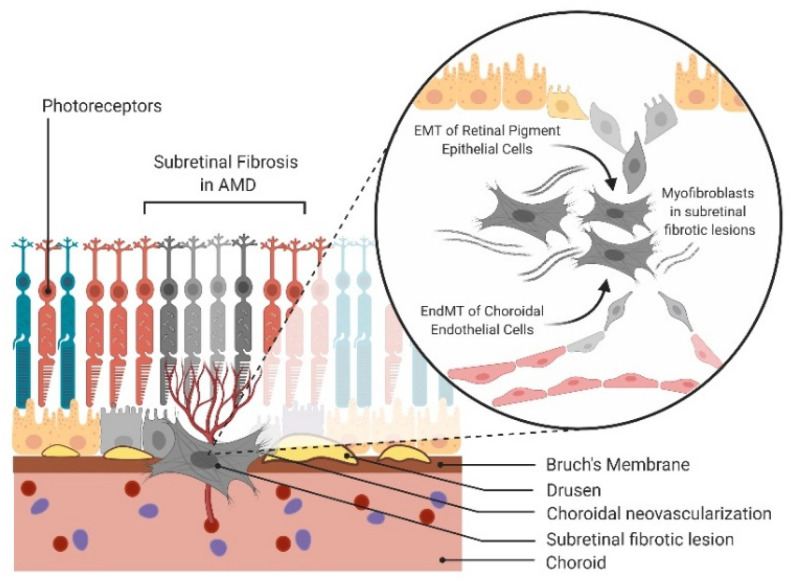
Epithelial-mesenchymal transition (EMT) and endothelial-mesenchymal transition (EndMT) in age-related macular degeneration (AMD). Schematic of EMT of retinal pigment epithelial (RPE) cells and EndMT of choroidal endothelial cells (CECs) contributing to the mesenchymal cell population in subretinal fibrosis of AMD.

In this review, we explore the roles of EMT and EndMT in the pathogenesis of AMD (Figure 1). We provide evidence supporting EMT and EndMT as integral processes in the progression of dry and wet AMD with a focus on signaling pathways, inflammation, oxidative stress, metabolism, mitochondrial and autophagic dysfunction. We discuss the synergistic activity of RPE and CECs and how this interaction may foment EMT and EndMT processes through a vicious cycle. We discuss whether EMT and EndMT contribute differently to the retinal wound healing process or whether they give rise to the same pool of mesenchymal cells. We conclude with a discussion on insightful directions for future research opportunities in reversing subretinal fibrosis through mesenchymal–epithelial or mesenchymal–endothelial transition (MET) to restore vision.

## 2. Definitions of EMT and EndMT

While EMT is a physiological process required for normal embryologic development, this process can be hijacked in pathological conditions to facilitate tissue fibrosis and cancer metastasis [11]. Thus, there are three categories of EMT: type 1 (embryology), type 2 (wound healing) and type 3 (cancer metastasis) [12]. The focus of this review is on type 2 EMT. Normally activated during tissue regeneration in wound healing and repair, abnormal activation of type 2 EMT in a chronic and excessive manner potentiates pathological tissue fibrosis. Initially discovered as a key mechanism in embryonic cardiac development, EndMT research has now grown to encompass all three types of EMT including fibrosis and cancer metastasis [13]. Importantly, EndMT is often considered a subcategory of EMT since the endothelium is a specialized type of epithelial cell specifically lining blood vessels.

Both EMT and EndMT follow a highly coordinated sequence of events. The first step in EMT involves downregulation of E-cadherin, a protein central to maintaining lateral contacts of neighboring epithelial cells through adherens junctions [3]. Increased expression of mesenchymal markers such as N-cadherin, vimentin, α-smooth muscle actin (α-SMA), ECM proteins (fibronectin and collagens), as well as ECM remodelling proteins (MMPs and TIMPs) are concomitant to this cellular transition into a mesenchymal phenotype [14].

An analogous process occurs in EndMT, where endothelial cells lose their endothelial markers, including vascular endothelial-cadherin (VE-cadherin), CD31, platelet-endothelial cell adhesion molecule 1 (PECAM-1), tyrosine kinase with immunoglobulin-like and EGF-like domains (TIE-1, TIE-2) and von Willebrand Factor (vWF) [15]. Additionally, these cells gain the same mesenchymal markers including N-cadherin, vimentin and α-SMA [15]. Aberrant angiogenic sprouting of endothelial cells leads to rapid remodeling of the basement membrane through MMPs and plasminogen activator-dependent proteolytic degradation of ECM components [16]. Such alterations of ECM organization within the subretinal space may further drive EMT and EndMT by altering homeostatic mechanochemical signaling [17] and promote TGFβ activation.

Ultimately, in both EMT and EndMT, a dramatic cytoskeletal reorganization results in an elongated, spindle-shaped morphology characteristic of newly formed migratory mesenchymal cells. While both RPE and endothelial cells are highly differentiated cells, their ability to undergo such drastic morphological changes reveals immense cellular plasticity. The initial descriptions of EMT and EndMT suggested that this conversion from epithelial or endothelial cell into mesenchymal cell was a permanent conversion. However, more recently, these processes have lost the presumptions of directionality and permanence, now reflecting the reversibility and spectrum of intermediary states concomitant to these processes [18,19]. Partial EMT or EndMT permits hybridization of the EMT or EndMT state, expressing both epithelial/endothelial and mesenchymal biomarkers concurrently. Whether and how partial EMT and EndMT plays a role in AMD is yet to be investigated, but research in cancer metastasis may lend a clue, showing that this transitional phenotype is metastable and highly plastic with the capacity to undergo both partial or total reversal of the process [19]. Notably, cancer metastasis is a type 3 EMT, whereas subretinal fibrosis is a type 2 EMT and thus, conclusions cannot be directly drawn without proper investigation in appropriate AMD models.

During angiogenic sprouting, it is important to note that while endothelial cells express many EndMT and ECM remodeling genes, they retain intercellular junctions and migrate as a connected sheet of cells rather than as individual cells [18]. An emerging concept in understanding collective cellular migration is “unjamming transition”, whereby epithelial cells move collectively and cooperatively. This work has been pioneered by the Fredberg group in bronchial epithelial cells as a model to understand lung fibrosis in asthma [20,21]. Differentiating parallels between unjamming transition and collective endothelial cell migration in ocular angiogenesis provides an exciting subject for future investigation.

## 3. TGFβ as the Master Regulator of both EMT and EndMT

Multiple extracellular ligands are involved in the initiation and progression of the EMT and EndMT programs [14]. Robustly studied for its central role in governance of EMT and more recently, EndMT, the ligand transforming growth factor-beta (TGFβ) is considered the master regulator of these processes [22]. Other ligands involved in EMT include hepatocyte growth factor (HGF), fibroblast growth factor (FGF), epidermal growth factor (EGF), connective tissue growth factor (CTGF), insulin-like growth factor-2 (IGF-2) and inflammatory mediators such as interleukin-1 (IL-1) [23]. In EndMT, other inducers include the inflammatory cytokines IL1-β, IL-6 and tumor necrosis factor-alpha (TNF-α) in addition to the widely studied TGFβ [22].

TGFβ belongs to the TGFβ superfamily of growth factors implicated in several physiological and pathological conditions. These span from embryological development to tumor metastasis, autoimmune diseases and fibrotic diseases of the eye [3,24]. TGFβ is a tightly regulated signaling molecule with complex transcriptional and translational processes. TGFβ is secreted from cells in a latent form, comprising a dimeric pro-peptide (the latency-associated peptide, LAP) and a latent TGFβ-binding protein (LTBP). Together, this tripartite complex of TGFβ, LAP and LTBP is termed the large latent complex [25]. The LAP confers latency while the LTBP functions to direct and sequester the growth factor into the ECM and assist in converting latent TGFβ into its active form. Sequestration of latent TGFβ in the ECM is essential for proper mobilization of the latent cytokine and its activation [26].

Latent TGFβ can be activated in various ways such as heat, ultraviolet radiation, acidic pH, proteolytic cleavage by MMPs, reactive oxygen species (ROS) and mechanical shear stress [27]. Once activated, TGFβ binds to specific transmembrane serine/threonine kinase receptors to transduce its intracellular signal by phosphorylating the canonical Smad signaling pathway. TGFβ can also activate an extensive host of non-canonical signaling pathways including the mitogen-activated protein kinase (MAPK; p38, JNK, ERK), phosphatidylinositol-3-kinase/Akt (PI3K/Akt), mammalian target of rapamycin (mTOR), Hippo/YAP, β-catenin/Wnt, protein kinase C and Rho-like GTPase that intricately modulate distinct downstream TGFβ responses [28].

Of the three described mammalian TGFβ isoforms (TGFβ1/2/3), all have been detected in the vitreous and aqueous humor of the human eye [29]. Approximately 90% of vitreal TGFβ is found in the latent form [30]. TGFβ2 (both the latent and activated form) is the predominant isoform in the eye, while TGFβ3 is present at low levels, and TGFβ1 is barely detectable in normal physiological conditions [31,32,33,34]. The ratio of the three active TGFβ isoforms is 2.5:1:0 for β2:β3:β1 respectively in human aqueous humor [31]. In the outer-retina, RPE constitutes the main source of TGFβ through a large production and secretion of TGFβ2, while the expression of TGFβ1 and TGFβ3 is negligible to null [35,36,37]. This makes the concentration of TGFβ2 in the RPE-Bruch’s membrane-choroid complex 10 times higher than levels normally observed in the neural retina [29]. Under physiological conditions, the presence of TGFβ2 in the subretinal space serves as a potent immunosuppressant to ensure ocular immune privilege, a special status in the eye to limit immune cell entry and inflammation to preserve vision [38]. However, in pathological ocular conditions, a significant increase in TGFβ2 secretion accompanies loss of cell–cell adhesions between RPE cells [37] and increased vitreal TGFβ2 has been observed in EMT of RPE and PVR models [29].

## 4. Evidence and Molecular Drivers of EMT/EndMT in AMD

Histological studies on CNV membranes excised from patients with nAMD show a complex array of cells and proteins including RPE, vascular endothelial cells, macrophages, pericytes, fibroblastic cells, myofibroblasts and ECM [39,40,41]. During AMD, RPE cells degenerate, lose their characteristic epithelial morphology and function, capacitating their migration into the retina and the sub-RPE space [4,42]. Closer histological inspection of these “degenerating” cells suggests that they may not be dying but rather, they may be reversibly transforming into mesenchymal cells via EMT to survive the harsh microenvironment during AMD disease progression [42,43,44].

A key family of transcription factors extensively studied in both EMT [45] and EndMT [46] is the Snail superfamily of zinc-finger transcription factors. The Snail superfamily, including the founding member, Snai1 (Snail) and later additions Snai2 (Slug) and Snai3 (Smuc), are key players in the transcriptional repression of E-cadherin [45] and VE-cadherin [47]. Increased immunoreactivity for mesenchymal markers (vimentin and Snai1) and reduced E-cadherin was found in RPE from donor dry AMD tissues, characteristic of a type 2 EMT response [48]. Surgically excised CNV lesions contain α-SMA-positive stromal cells believed to be transdifferentiated RPE [40]. Upregulated expression of the EMT transcription factor, Snai1 was localized in RPE nuclei in CNV donor tissues from wet AMD patients [49]. The importance of Snai1 has been validated in vitro by blocking EMT of RPE under hypoxic conditions via silencing of Snail and TGFβ2 using a human RPE cell line, ARPE-19 [50].

Snai1 is also prominently described for its key role in angiogenesis. Snai1 is expressed on sprouting vessels under both physiological conditions in the developing retinal vasculature and in pathological angiogenic models of laser photocoagulation-induced CNV and oxygen-induced retinopathy (OIR) mouse models [5]. Snai1 overexpression in human umbilical vascular endothelial cells (HUVECs) induced cellular elongation and enhanced cell motility with lamellipodia formation, whereas Snai1 knockdown blocked migration, invasion and sprouting. RNA sequencing analysis showed that Snai1 knockdown reduces expression of genes governing cytoskeletal arrangement and ECM remodelling [5]. Moreover, intravitreal injection of small interfering RNA (siRNA) of Snai1 suppressed new vessel formation in the developing retina and in mouse models of laser-induced CNV and oxygen-induced retinopathy [5]. Activation of endothelial cells is an important initial stage in angiogenic sprouting, whereby endothelial cells are required to generate a highly invasive phenotype much like the process of EndMT. Endothelial cells must undergo a drastic transformation through cell–cell and cell–matrix contact reconstruction, ECM degradation/synthesis, and migratory activity, as well as lamellipodia and filopodia formation, to ultimately generate new blood vessels [18]. While further investigation is required to unravel the link between EndMT and ocular angiogenesis, it is clear that Snai1, an EndMT transcription factor plays an important role in promoting the early phase of ocular angiogenesis including CNV development [5].

### 4.1. Role of Cytokine-Mediated Signaling Pathways

**Figure 2:**
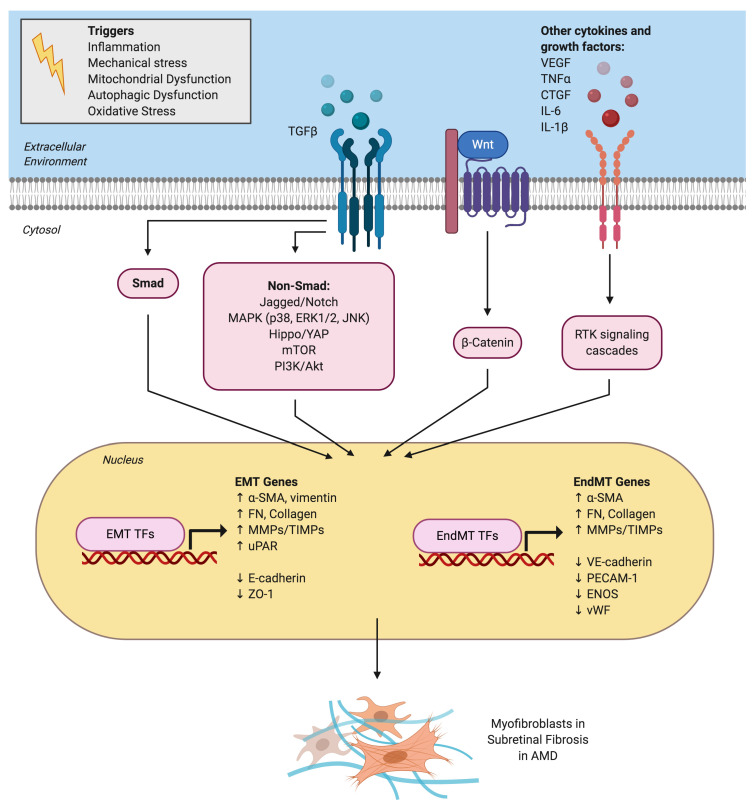
Signaling pathways and mechanistic drivers of epithelial-mesenchymal transition (EMT) and endothelial-mesenchymal transition (EndMT) in age-related macular degeneration (AMD). Various extracellular cytokines and receptor tyrosine kinase (RTK) signaling pathways are involved in activating EMT of retinal pigment epithelial cells and EndMT of choroidal endothelial cells. TGFβ is the master regulator and activates both the canonical Smad signaling pathway and a host of non-canonical signaling pathways. The Wnt/β-catenin signaling pathway also plays a key role in both EMT and EndMT. Activation of EMT and EndMT results in the upregulation of various transcription factors (TFs) such as Snai1 and mesenchymal genes and the downregulation of epithelial or endothelial genes, respectively. This ultimately leads to myofibroblast transdifferentiation and excessive extracellular matrix deposition observed in subretinal fibrotic lesions in AMD.

Both EMT and EndMT are regulated through a diverse network of signaling pathways such as the canonical Smad and non-canonical TGFβ signaling pathways [51] (Figure 2). Critical to the pathogenesis of CNV is the process of RPE detachment and dissociation. Disruption of RPE cell–cell contact is required for TGFβ to initiate the EMT program [52]. Similarly, disruption of vascular endothelial cell–cell contact is required for initiation of the EndMT program in models of organ fibrosis [53] but is yet to be explored specifically for CECs in AMD. Junctional complexes such as adherens junctions and tight junctions are crucial in maintaining structural integrity, apicobasal polarity and barrier function of both epithelial [54] and endothelial cells [55]. These complexes are localized at the plasma membrane and disrupted upon activation of EMT and EndMT signaling [13]. Disruption of cadherins, the integral protein of adherens junctions (E-cadherin or P-cadherin for epithelial cells and VE-cadherin for vascular endothelial cells) is an initiating factor in loss of cell–cell adhesion.

β-catenin is sequestered and maintained by cadherins at adherens junctional complexes [56]. However, once EMT or EndMT is activated, β-catenin takes on a new role as a signaling molecule for the Wnt pathway as it disassociates from the cadherins at the membrane, translocates to the nucleus and binds to the TCF/LEF family of transcription factors to activate EMT and EndMT transcription factors including Snai1 [57,58]. Computational modeling of transcription factors regulating EMT in RPE identified lymphoid enhancer-binding factor 1 (LEF-1) as one of the candidate nodes in the EMT transcriptional regulatory network of RPE [59].

Nuclear localization of β-catenin has been observed in RPE cells undergoing EMT and inhibition of β-catenin signaling prevented both EMT and proliferation [60,61]. Binding of cadherins to the cell surface also activates a cascade of protein kinases including the Hippo signaling pathway [62] and Src family kinases [63,64]. Blockade of Src kinases prevented EMT of RPE in vitro by maintaining adherens junctional integrity [64]. The EMT gene, Zeb1, is a known target of the Hippo transcription factors, Yap and Taz. While Yap was not detectable in primary mouse RPE cells, Taz translocated to the nucleus during EMT of RPE through activation of Zeb1 via the Taz-Tead complex [65].

The Jagged/Notch pathway has also been implicated in both EMT and EndMT. During TGFβ2-induced EMT in RPE, a concomitant upregulation of Jagged-1, Notch-3 and their downstream target genes Hes-1 and Hey-1 was observed [66]. Knockdown of Jagged-1 or treatment with DAPT, a specific inhibitor of Notch receptor cleavage, blocked TGFβ2-induced EMT in RPE by suppressing expression of Snail, Slug and Zeb1. Overexpression of Jagged-1 induced EMT in RPE like that of TGFβ2. TGFβ2-induced upregulation of Jagged-1 in RPE was associated with activation of the canonical Smad signaling pathway and non-canonical PI3K/Akt and MAPK pathways. A complex interplay between ERK1/2, Smad and Jagged/Notch has also been reported in TGFβ2-induced EMT in RPE [67]. Intriguingly, inhibition of p38 MAPK but not ERK1/2 blocked the increase in type 1 collagen (COL1A1 and COL1A2) expression and transcriptional activity induced by TGFβ2 in ARPE-19 [68]. Further support for the role of p38 MAPK in EMT of RPE is highlighted in whole transcriptome RNA sequencing of human PVR membranes and inhibition of p38 signaling effectively suppressed EMT of RPE induced by the synergistic activity of TGFβ1 and TNFα [69].

TGFβ pathway activators were highlighted in a transcriptome wide expression profile analysis in a RPE EMT model [4]. Many RPE wound response genes showed altered expression in wet and dry AMD including key members of the TGFβ family. Notably, there was no representation of the TGFβ pathway genes in the RPE-choroid GA data set [4]. This may be because GA is a disease state best defined by cell death, where the actively involved cells are possibly restricted to the margins of the diseased area. However, the TGFβ pathway was highly overexpressed in the CNV and GA data set, highlighting the importance of the RPE wound response in advanced AMD [4]. This elevated TGFβ expression in AMD corroborates data from a collaborative genome-wide association study that links the TGFβ receptor type I (TGFBR1) polymorphism with risk of developing AMD [70].

A driving force for TGFβ in nAMD is inferred by animal models showing that TGFβ inhibition effectively blocks CNV formation [71,72] and subretinal fibrosis [73]. The pro-angiogenic effects of TGFβ are further shown in its capacity to upregulate the expression of vascular endothelial growth factor-A (VEGF-A) in RPE through MAPK signaling [73]. Despite clear evidence of increased vitreal and retinal TGFβ levels in clinical [70] and experimental CNV [74], its role in pathological angiogenesis remains controversial. Increasing evidence shows that TGFβ can exert anti-angiogenic functions. Surprisingly, the concentration and activity of TGFβ was found to be downregulated in the aqueous humor of patients with nAMD [31]. This discrepancy may be linked to the fact that clinical samples were taken from nAMD patients who were actively undergoing anti-VEGF therapy, which may impact on TGFβ levels. Thus, the potential of TGFβ inhibitors to treat AMD should be met with caution until further research is conducted to fully characterize the role of TGFβ in AMD. It is possible that TGFβ may play different roles during the early, intermediate and late stages of AMD. The dual role of TGFβ has been extensively studied in cancer metastasis with reports of acting as a tumor suppressor in normal cells and early cancer development but as the tumors progress, the protective effects of TGFβ are lost and instead, it switches to promote cancer invasion and tumor metastasis [75]. Currently, the role of TGFβ in different stages of AMD is unclear and opens an important area of future investigation.

Besides TGFβ, other growth factors also play a key role in the progression from neovascularization into fibrosis including VEGF and connective tissue growth factor (CTGF). The ratio of VEGF and CTGF levels in the retina drive a so-called “angio-fibrotic switch”, whereby increased levels of CTGF sequester VEGF. When the levels of CTGF sufficiently overcome VEGF levels, angiogenesis ceases and excess CTGF drives scar formation [76]. CTGF correlates positively and VEGF correlates negatively with the level of fibrosis. When the balance between these two factors shifts to a specific threshold ratio, an angio-fibrotic switch occurs, and fibrosis ensues [77]. While CTGF itself does not directly affect neovascularization, it may indirectly modulate VEGF levels [78,79]. In order to reduce fibrosis-associated vision loss, supplementing anti-VEGF therapy with anti-CTGF targeting agents may be required.

Targeting CTGF, a more specific downstream regulator of the pro-fibrotic activity of TGFβ may be a more feasible therapeutic option for eradicating fibrosis. In a rat model of diabetic retinopathy, Hu et al. (2014) combined anti-VEGF (bevacizumab) and anti-CTGF therapy (using CTGF shRNA) and showed that this dual-target intervention was more effective in improving microvessel ultrastructure compared to single-target intervention [80]. While the angio-fibrotic switch has been extensively studied in proliferative diabetic retinopathy where retinal neovascular membranes convert to retinal fibrosis, a similar underlying process may be extrapolated to AMD and guide future research in understanding the interplay of growth factors mediating CNV development and regression into subretinal fibrosis.

### 4.2. Role of Inflammation in EMT/EndMT During AMD

The transition from early to advanced AMD has many features consistent with an aberrant wound healing response resulting from underlying degeneration, oxidative stress and chronic inflammation. Drusen disrupts the normal retinal tissue architecture, impedes transport between the RPE and choroid and serves as sites of activation of the complement signaling cascade [81,82]. Immunohistochemical staining for NLR Family Pyrin Domain Containing 3 (NLRP3) is found in RPE of patients with advanced AMD, suggesting inflammasome activation in AMD pathogenesis [83,84]. Inhibition of the interleukin-6 (IL-6) receptor suppressed a mouse model of subretinal fibrosis [85]. Injection of activated macrophages into the subretinal space induced EMT in RPE, establishing a novel animal model of focal subretinal fibrosis [86]. Overexpression of miR-194 suppressed TGFβ1-induced EMT in APRE-19 cells by functionally targeting ZEB1 and inflammatory pathways [87]. The anti-inflammatory drug, NS-398 (COX-2-selective antagonist), blocked CNV in subretinal fibrosis with reduced macrophage infiltration, reducing both VEGF and TGFβ expression in the RPE-choroid complex [88]. Another drug with anti-inflammatory properties, resveratrol, also effectively suppressed TGFβ2-induced EMT in RPE [89].

Pro-inflammatory cytokines can propagate their signals by activating endothelial cells into mesenchymal cells through EndMT [53]. Two such pro-inflammatory cytokines, TNFα and interleukin-1β induced human retinal microvascular endothelial cells to undergo EndMT with loss of endothelial cell markers (VE-cadherin and endothelial nitric oxide synthase) and gain of mesenchymal markers (Snai1, transgelin, calponin and fibroblast specific protein-1) [10]. Further investigation is needed to validate and generalize these observations to determine whether similar mechanisms also exist for CECs.

### 4.3. Metabolic Dysfunction and Autophagy in AMD

As described above, abnormal RPE cells in AMD have morphologic features characteristic of a type 2 EMT, a process that can be activated by oxidative stress-induced impairment of autophagy and lysosomal function [90,91,92]. EMT can be considered a transcriptional program, allowing cells to survive stresses including oxidative stress but at the cost of losing their specialized functions as epithelial cells. Under homeostatic conditions, cells (RPE and neurons) within the macular region are exposed to constant substantial oxidative and metabolic stress [93] and as such, mitochondrial dysfunction has emerged as a key mediator in the pathogenesis of AMD [94]. Reduced mitochondrial number [95], preferential mtDNA damage [96,97], higher levels of mtDNA rearrangements [98,99], decreased total ATP synthase subunits [100,101] and decreased mitochondrial heat shock protein (mtHsp70) [100] have been reported in RPE from AMD donors compared to control subjects. Reduced mitochondrial bioenergetic function [102] and reduced ATP production [103] are evident in RPE cells isolated from human donors with AMD compared to healthy controls. Moreover, defects in the major energy sensor, AMP-activated protein kinase (AMPK), have been implicated in EMT of RPE [104]. Enhancing mitochondrial respiration using dichloroacetate, a structural analogue of pyruvate, blocked TGFβ2-induced EMT in RPE [105]. High glucose induces both EMT [106] and EndMT [107] in RPE and retinal endothelial cells, respectively, implicating the role of hyperglycaemia-induced metabolic dysfunction.

Mechanistic insights into mitochondrial dysfunction in AMD have highlighted a protective role for proliferator-activated receptor gamma coactivator 1-alpha (PGC-1α) in RPE during AMD [94,108]. Repression of PGC-1α in mice and exposure to a high-fat diet resulted in AMD-like abnormalities in the RPE [109]. Work in our laboratory identified PGC-1α as a master regulator of mitochondrial biogenesis and function in RPE, increasing in expression with RPE maturation [110]. Recently, we showed that silencing PGC-1α in ARPE-19 profoundly disrupted mitochondrial function, redox state, energy sensor activity and autophagic function [111]. Intriguingly, we discovered that silencing PGC-1α in RPE ultimately induced an EMT response [111]. Our laboratory identified ZLN005 as a selective PGC-1α transcriptional regulator in enhancing mitochondrial respiratory function in RPE and protecting RPE from oxidative stress [112]. Investigations into whether ZLN005 may also block EMT in RPE are currently underway in our laboratory.

Lysosomal dysfunction has been identified as a major pathogenic process in AMD [113] and may also drive RPE into EMT to support cell survival in a stressful microenvironment. βA3/A1-crystallin, encoded by the *Cryba1* gene, is an important protein for lysosomal clearance in RPE [48]. Age-dependent lysosomal deficiency has been implicated in numerous age-related diseases such as AMD as well as Parkinson’s and Huntington’s diseases [114]. Genetically engineered mouse models with a loss-of-function mutation in *Cryba1* showed an AMD-like phenotype and also expressed key molecular markers of EMT [48]. Autophagy guides the degradation of unwanted or dysfunctional cellular components by delivering them to lysosomes. Reduced autophagic capacity has been linked to AMD [115]. Defects in mitophagy, a selective form of autophagy that specifically removes dysfunctional mitochondria from cells has also been implicated in AMD pathogenesis [116]. In cancer studies, the activation of autophagy, mitophagy and impaired mitochondrial functionality have been linked to both EMT [117] and EndMT [118], warranting further research into whether parallels exist for RPE and CECs.

## 5. Role of the Extracellular Matrix in AMD-Associated EMT/EndMT

Sandwiched between the RPE and choriocapillaris is Bruch’s membrane, a pentalaminar structure consisting of elastin- and collagen-rich ECM. Bruch’s membrane acts as a molecular sieve to regulate the reciprocal exchange of biomolecules, nutrients, oxygen and metabolic waste products between the retina and the general circulation. Since Bruch’s membrane is acellular, transport occurs primarily via passive diffusion and depends on the hydrostatic pressure on either side of the membrane. On the photoreceptor side of the RPE, the subretinal space is occupied by the interphotoreceptor matrix, a highly organized, hydrophilic matrix composed of large glycoproteins and proteoglycans that play a key role in retinal adhesion to the RPE and regulate nutrient transport [119].

Due to its anatomical position and functional role in retinal homeostasis, the significance of Bruch’s membrane cannot be overlooked in AMD pathogenesis. The ECM acts as a supportive framework for RPE and CECs, creating an internal environment for signal transduction, nutrient transport, metabolism, structural integrity and scaffolding to regulate cellular adhesion, migration, proliferation and differentiation. A physiological balance exists between the synthesis and degradation of ECM components and any disruption of this homeostasis can initiate and propagate disease states. As a result of CNV, vessels from the choroid proliferate and penetrate through the ECM border, their immature vascular walls inducing an increase in leaks of serum, lipoproteins and hemorrhage into the extracellular space.

### 5.1. ECM Remodelling During AMD Progression

One key event during EMT and EndMT is aberrant ECM remodeling and mounting evidence suggests that age- and/or disease-associated alterations in ECM composition act as driving forces of EMT and EndMT. RPE degeneration is preceded by age-dependent changes in Bruch’s membrane [120,121], such as increased thickness, reduced permeability, and accumulation of lipids, extracellular material, local oxidation and glycation products [122,123,124,125]. This suggests that alterations of Bruch’s membrane may be partly responsible for the subsequent RPE dysfunction.

This concept is supported by in vitro analysis showing that the culture of normal human RPE on Bruch’s membrane collected from aged or AMD patients drastically changes their behavior and gene expression profiles [126,127,128]. While cobblestone RPE have been successfully cultured on human submacular Bruch’s membrane explants with an intact RPE basement membrane [129], attempts to grow RPE on the deeper portion of the inner collagenous layer or elastic layer of Bruch’s membrane have been less successful [129,130]. This may explain why patients who undergo submacular surgery with CNV excision have poor visual recovery [131]. Proliferation of cobblestone RPE monolayers are also reduced if RPE are grown on older donor Bruch’s membranes derived from AMD patients [128,130]. This lack of adhesion may also be explained by age-related deposits of anti-adhesive molecules and reduced integrin ligands that typically promote RPE attachment [132].

Further ECM alterations occur during both early and advanced stages of AMD as an imbalance of MMPs and TIMPs enhance pathological production, accumulation and degradation of ECM proteins by RPE and CECs [133]. The most prominent ECM components in subretinal fibrosis are collagen types I and IV and fibronectin, with small amounts of collagen types III, V and VI [134]. Collagen type IV surrounding RPE in the stroma is also a major component of the basal membrane of normal RPE. Subretinal neovascular membranes are characterized by large “feeder” vessels with many new capillaries in different stages of maturation, embedded in an abundant stroma. Within the lesion, RPE-like pigmented cells and fibroblast-like cells form most of the non-vascular cell types. As the new vessels sprout from the choroid, they induce a splitting between the RPE cells and Bruch’s membrane, notably this only applies to type 2 CNV due to its anatomical location [6]. The newly formed capillaries have morphologically ill-defined basement membranes that contain substantial levels of collagen type IV and fibronectin, but unlike normal capillaries, lack laminin or heparan sulfate proteoglycans [134].

### 5.2. TGFβ and ECM Changes in AMD

As noted, imbalances in MMP-2/9 and TIMP-1/2 in dysfunctional RPE play a key role in both early dry AMD and advanced wet AMD [133,135]. MMP-2/9 digest the primary structural ECM proteins including fibronectin, collagen IV, collagen V and laminin. Loss of MMP-2 leads to an accumulation of collagen IV that manifests as deposits underneath the RPE layer. Indeed, MMP-2 is the most abundant enzyme synthesized by RPE cells and disordered MMP-2 activity is a key pathogenic factor in early AMD development [136,137]. MMP-1 degrades collagen I-III and its decreased activity favors soft drusen development. Activation of MMP-1 by lysosomal enzymes in aged and dysfunctional RPE cells leads to the development of advanced wet AMD in susceptible individuals. Increased MMP levels have been reported in CNV membranes [138,139]. Exogenous TGFβ can stimulate the release of MMP-9 by retinal capillary endothelial cells as can direct contact with astrocytes or Müller cells [140]. TGFβ increases permeability of bovine retinal endothelial cells by a mechanism that appears to involve production of MMP-9 [140]. Evidently, MMPs play a key role in mediating the pathogenesis of AMD and indeed, the efficacy of MMP inhibitors has shown promise in treating CNV [138].

### 5.3. uPA and ECM Changes in AMD

Another family of proteolytic enzymes, namely the urokinase-type plasminogen activator (uPA) and its receptor (uPAR) have been implicated in ECM remodeling during advanced nAMD [141]. uPA is a serine protease that binds with high affinity to its cell surface receptor, uPAR, thus stimulating the interaction between uPAR and transmembrane proteins such as integrins to regulate cytoskeletal reorganization, cell migration, differentiation and proliferation. Both TGFβ1 [142] and TGFβ2 [143] increased the expression of uPAR in RPE. Clinically, a visually devastating complication of nAMD is the development of submacular haemorrhage [144] and may provide a potential source of blood clotting factors such as uPA enzymes.

Plasminogen activator inhibitor type-1 (PAI-1) is the primary endogenous inhibitor of uPA. PAI-1 is expressed in human CNV specimens [145] and was localized to migrating endothelial cells [146]. Increases in PAI-1 have been localized to newly forming retinal vessels in a laser-induced CNV model [145] and an OIR mouse model [147]. In RPE, increases in PAI-1 occur concomitantly with the mesenchymal marker, α-SMA, in a model of sphingosine-1-phosphate (S1P)-induced EMT [148]. In contrast, a rat model of OIR showed that high doses of recombinant PAI-1 could block new retinal vessel formation [149]. This may be explained by the dual and dose-dependent role of PAI-1 in angiogenesis with PAI-1 exerting pro-angiogenic effects at low concentrations and anti-angiogenic activity at high concentrations [145,150]. This suggests that an optimal physiological level of PAI-1 may be required to ensure homeostasis of retinal vascular health.

### 5.4. Mechanotransduction in AMD

Altered mechanical properties of the ECM such as matrix stiffness can drive fibrosis [151]. Cells possess the ability to sense their physical surroundings and convert mechanical cues into biochemical signals triggering downstream intracellular events, a process termed mechanotransduction [152]. Integrins and integrin-linked kinases are responsible for physically anchoring cells to the ECM of their underlying basement membrane and in doing so, serve as bidirectional hubs transmitting biomechanical signals between cells and their microenvironment [153]. In surgically excised human CNV specimens, expression of integrins αvβ3, α1β1, α2β1 and α5β1 colocalized with endothelial cells in tissues obtained from early to mid-stages of AMD [154]. Application of tensile forces to cultured RPE cells using collagen-coated magnetite beads and magnetic fields was sufficient to upregulate EMT markers (MMP-2 and fibronectin) and robustly activated p38 MAPK signaling [155]. The mechanosensitive ion channel, transient receptor potential vanilloid 4 (TRPV4), is known to regulate matrix stiffness and mechanosensing in EMT in models of skin fibrosis [156], breast cancer [157] and corneal fibrosis [158]. It is unknown whether TRPV4 also plays a role in EMT and EndMT in AMD, but it is promising to note that this mechanosensitive channel is expressed in RPE cells [159] as well as both retinal and choroidal endothelial cells [160].

Clinically, excessive mechanical stress can be induced by the presence of epiretinal membranes (ERM) that may complicate AMD. ERM, a fibrocellular membrane that proliferates along the inner retinal surface at the macular region can exert tractional forces that lead to macular folds, macular edema and in advanced cases, foveal detachment [161]. Co-existence of an ERM has been observed in 26% of eyes with nAMD [162]. Co-treatment of adult human RPE stem cells with TGFβ and TNFα synergistically activated an EMT program, producing fibroblastic and contractile membranes resembling ERM. Treatment of RPE cell suspensions with vitreous obtained from ERM patients induced EMT following mechanical stress by cell scraping [163].

## 6. Therapeutic Considerations: Modulating Microenvironmental Signals

While extensive literature has investigated the roles of RPE and CECs in AMD separately, the in vivo situation is far more complex. In the following section, we discuss how the microenvironment may influence the processes of EMT and EndMT in vivo through synergistic effects between RPE and CECs. We also discuss the promise of reversal and inhibition of EMT and EndMT as a therapeutic approach for AMD.

### 6.1. Synergistic Interaction between RPE and CECs

During development, the RPE controls formation of the choriocapillaris and later its maintenance during adulthood. Development of the choroidal vasculature depends on proper RPE differentiation. The crucial relationship between RPE and CECs is not restricted to embryology but persists into adulthood. Histopathological studies in atrophic AMD patients shows that early damage to the RPE layer precedes atrophy of the choriocapillaris indicating that choriocapillaris loss is secondary to RPE dysfunction [164,165,166]. When RPE are removed from Bruch’s membrane, degeneration of the choriocapillaris was observed, suggesting that RPE may produce and release vital tissue-specific angiocrine factors [167,168,169].

In both clinical and experimental settings, choroidal atrophy and/or ischemia is associated with corresponding regional RPE lesions and serous detachment [170,171]. Certainly, reduction in vascularity inevitably leads to reduced oxygen, nutrients and circulating factors, hence altering cell function and survival of surrounding tissues. However, in vitro experiments have also identified a synergistic interaction between RPE and endothelial cells in enhancing the differentiation and functionality of each cell type through the exchange of homeostatic paracrine factors [172,173]. Ocular overexpression of TGFβ1 induced EMT in RPE and subsequent atrophy of the choriocapillaris [174]. Modulation of RPE ECM deposition also plays a role in mediating the heterotypic trophic effects [172]. RPE integrin receptors can sense these changes and trigger Rho GTPase signals to enhance RPE barrier function and RPE tight junction formation [173]. Given this synergistic relationship between RPE and CECs, a currently unanswered question is whether similar synergistic effects exist between EMT and EndMT in the development of subretinal fibrosis.

One potential unifying target is oxidized lipoproteins that act on both RPE and CECs. Oxidized low density lipoproteins (OxLDL) are known to preferentially accumulate in the macular region and are important players in AMD pathogenesis [175]. OxLDL is a key contributor to inflammation and oxidative stress in AMD by increasing ROS accumulation [176] and activating the NRLP3 inflammasome in RPE [177], thus promoting RPE senescence and death [178]. Additionally, OxLDL enhances CNV lesions by inducing EndMT in Rhesus monkey choroid-retinal vascular endothelial cells (RF/6A) through the TGFβ2/Smad signaling axis [179].

Whether a similar crosstalk between RPE and CECs is involved in AMD progression by potentiating EMT or EndMT is unclear. Few studies have explored whether any factors are released by these cells to support the mesenchymal transdifferentiation of the other cell type. A recent study demonstrated that exosomes released from EMT-induced RPE cells are enriched in pro-angiogenic factors and promote endothelial cell migration and tube formation [180]. Whether similar pro-EMT signaling factors are released by CECs during early AMD is unknown. However, evidence suggests that RPE can produce thrombin from serum-derived prothrombin and that thrombin can act as a potent inducer of EMT by RPE via autocrine activation of platelet-derived growth factor (PDGF)-receptor signaling [181]. Thrombin reduced zonula occludens (ZO)-1 gene expression and increased expression of mesenchymal markers, α-SMA and the pro-alpha1 chain of collagen type I, indicating an EMT response. Taken together, these data highlight that the coagulation cascade may facilitate EMT of RPE and contribute to subretinal fibrotic membrane formation.

Once successfully restored, an organized and functional RPE monolayer has the capacity to produce a Bruch’s membrane-like matrix [182]. ARPE-19 cells cultured in the presence of dextran sulphate to promote ECM secretion produced proteins found in the inner layers of Bruch’s membrane such as fibronectin, vitronectin, collagen IV, collagen V and laminin-alpha-5 [182]. Proteins such as elastin (found in the middle elastic layer) or the outer layers (collagen VI) were not produced by ARPE-19 [182]. Since the elastic layer of Bruch’s membrane is prone to calcification with age [183] and breakdown of elastic fibers can potentially release pro-angiogenic proteins [184], the lack of elastin production by RPE in this model may be beneficial. This is a promising finding for future EMT-targeting therapies as restoration of the epithelial phenotype may have the potential to also restore a healthy microenvironment, thus ensuring long-term drug efficacy.

### 6.2. Reversal of EMT and EndMT as a Potential Therapeutic Approach for AMD

Reversal of EMT and EndMT is known as mesenchymal–epithelial transition or mesenchymal–endothelial transition, herein collectively termed as MET. Ambitious as it may sound, activation of the MET program and restoration of a functional monolayer of epithelial or endothelial cells is a plausible therapeutic approach for combating AMD. In vitro studies show that RPE have the capacity to undergo MET depending on the culture conditions. High plating density, low passage number and reduced serum concentration are effective in limiting cell spreading and proliferation, thus re-establishing the epithelial monolayer [185,186,187].

Activation of EMT, EndMT, and MET are conserved embryological processes and as much as EMT and EndMT can be hijacked by pathological cells, so too can we harness the MET program to restore the epithelial or endothelial phenotype. Whether MET induces cells to return to their innate cell type or whether it can be induced to differentiate into another cell type based on the microenvironment is currently unknown. Complete reversal of TGFβ-induced EMT in renal tubular epithelial cells required inhibition of both Zeb expression and the Rho signaling pathway [188]. Reversal of EndMT has been successfully accomplished in a brain microvascular endothelial cell line using hydrocortisone, which enhanced endothelial cell adhesive properties and barrier function [189]. MET remains an exciting area in AMD research, warranting further investigation.

If reversal of EMT is to be a feasible therapeutic strategy for combating AMD, then measures to restore the microenvironment may also be required. Without a proper substrate to attach to, RPE cells undergo apoptosis [190], an important hurdle for the success of single cell transplantation approaches. The fate of RPE cells seeded onto Bruch’s membrane depends on the composition and three-dimensional arrangement of the ultrastructural layer of the basement membrane available for reattachment. Age-related alterations in the biochemical and matrix composition of ECM molecules in the basement membrane may not only hinder proper RPE reattachment but could also limit the efficiency of MET strategies. Prior studies have evaluated the effect of rejuvenating Bruch’s membrane to enhance cell reattachment and survival and found that reengineering the surface of Bruch’s membrane explants either by cleaning and/or adding an ECM protein coating improves the final surface coverage of transplanted RPE [191]. Cleaning the surface of Bruch’s membrane by abrasive mechanical debridement promotes RPE repopulation [192]. However, adding ECM ligands to the elastin layers of Bruch’s membrane does not increase RPE attachment indicating that there are limitations to how many layers of Bruch’s membrane need to be intact in order for chemical surfacing to be feasible [193]. Whether such a strategy could be utilized to potentiate MET of endogenous RPE cells needs further investigation.

### 6.3. EMT and EndMT Inhibition

The inhibition of EMT and EndMT has primarily been explored in mechanistic experiments using in vitro cell culture and in vivo animal models with few drug candidates progressing to preclinical settings for AMD. Rather, most of the preclinical testing on blocking EMT of RPE has been pioneered in PVR. Methotrexate, a commonly used anti-cancer agent that inhibits de novo nucleotide synthesis, can also be used for its anti-inflammatory properties at low concentrations to treat rheumatoid arthritis and psoriasis [194]. Intraocular methotrexate is used to treat many inflammatory ocular conditions including uveitis, episcleritis, scleritis and sympathetic ophthalmia and at higher doses, primary intraocular lymphoma [195,196]. A small, retrospective pilot study on 29 patients with PVR or at high risk of PVR showed that intravitreal methotrexate infusion during surgery improved patient outcomes with stable visual acuity at 6 months in 83% of patients and low rates of recurrent PVR (20%) [197]. ADX-2191 (intravitreal methotrexate 0.8%) is currently in a phase 3 clinical trial for prevention of PVR, known as the GUARD trial [198]. Case reports on the use of intravitreal methotrexate in two nAMD patients who were unresponsive to anti-VEGF treatment showed promise with both patients exhibiting reduced subretinal fluid accumulation and perifoveal leakage [199]. Larger scale and longer-term clinical trials are required to evaluate the efficacy of methotrexate in AMD. Another promising drug is Ro5-3335, an inhibitor of runt-related transcription factor 1 (RUNX1), known to be upregulated during ocular angiogenesis [200] and is currently being tested for its efficacy in blocking PVR.

While EMT is a key process in both AMD and PVR, it is important to note that the two conditions represent vastly different timelines with AMD being a chronic condition that progresses at a slower rate compared to PVR that develops as a more acute response to the retinal trauma inflicted upon surgical repair of retinal detachment. Since subretinal fibrosis occurs in the end-stage of AMD, any potential intervention for this condition may need to be administered prophylactically over the course of a long period of time before the development of major visual symptoms, thus raising concerns regarding patient compliance and side effects.

## 7. Concluding Remarks and Future Directions

Extensive literature conclusively supports the importance of EMT and EndMT in AMD from in vitro experiments on RPE and ECs to in vivo animal studies and histopathological analyses of human AMD donor tissues. EndMT is still a relatively newly recognized type of cellular transdifferentiation and although it has been well accepted in the field of embryology, there has been some hesitation and controversy amongst the scientific community in recognizing EndMT as a pathogenic factor in pathological conditions in adult tissues. We hope that this review highlights the emerging involvement of both EMT and EndMT in AMD and its acceptance into the mainstream understanding of AMD pathogenesis. Many questions remain unanswered that will need to be addressed in future investigations, including the differential contributions of EMT and EndMT to the mesenchymal cell population in subretinal fibrosis, any potential synergistic activity between EMT and EndMT in AMD and the controversial dual role of TGFβ as both a pro- and anti-angiogenic factor in different clinical stages of AMD.

Another key issue is the role of other cells in giving rise to the mesenchymal cell population in subretinal fibrotic membranes in AMD. In an experimental CNV model using irradiated mice engrafted with green fluorescent protein (GFP)-positive bone marrow cells, many of the α-SMA-positive cells were also GFP-positive suggesting a systemic source of mesenchymal cells in CNV lesions [201]. Macrophages may also give rise to myofibroblasts via macrophage-myofibroblast transition (MMT) in subretinal fibrosis secondary to nAMD [202]. Choroidal pericytes may represent another potential source of myofibroblast precursors where they have been found to infiltrate CNV and transdifferentiate into collagen I-expressing cells in a photocoagulation CNV mouse model [203].

In order to adequately identify the cellular origins of the mesenchymal cell population, lineage tracing studies and genetic cell labeling need to be utilized to enhance our understanding. Tracking EMT, EndMT and MET in vivo in humans may also present an exciting possibility through improvement of positron emission tomography-magnetic resonance imaging (PET-MRI). The high resolution achieved through fluorodeoxyglucose-PET (FDG-PET) enables cell-tracing studies for a deeper analysis of fibrotic lesions as they develop in real-time.

To date, the literature exploring EMT and EndMT has largely studied these processes in isolation. While it is important to narrow our focus to gain mechanistic insights in EMT and EndMT, it is equally important to understand their synergistic potential in vivo. The mesenchymal cell population in subretinal fibrotic lesions requires further characterization to determine their cellular source, but also to determine whether mesenchymal cells derived from EMT or EndMT are distinct and contribute uniquely to different stages of AMD pathogenesis. Excavating the missing pieces of the puzzle is essential in harnessing the power of MET. We are optimistic that this review will encourage further investigation into the burgeoning field of EMT and EndMT in AMD and thereby, enable the development of novel drugs to combat AMD.

## Abbreviations

AMD	age-related macular degeneration
AMPK	adenosine monophosphate-activated protein kinase
α-SMA	alpha-smooth muscle actin
CEC	choroidal endothelial cell
COL1A1	collagen type I alpha 1 chain
CNV	choroidal neovascularization
CTGF	connective tissue growth factor
EC	endothelial cell
ECM	extracellular matrix
EGF	epidermal growth factor
EMT	epithelial–mesenchymal transition
EndMT	endothelial–mesenchymal transition
ENOS	endothelial nitric oxide synthase
ERK	extracellular-signal-regulated kinases
ERM	epiretinal membrane
FAK	focal adhesion kinase
FDG-PET	fluorodeoxyglucose- positron emission tomography
FGF	fibroblast growth factor
FN	fibronectin
GA	geographic atrophy
GFP	green fluorescent protein
HGF	hepatocyte growth factor
HUVECs	human umbilical vein endothelial cells
ICAM	intercellular adhesion molecule
IGF	insulin-like growth factor
IL	interleukin
LAP	latency associated peptide
LTBP	latent TGFβ-binding protein
MAPK	mitogen-activated protein kinase
MMPs	matrix metalloproteinase
MMT	macrophage-myofibroblast transition
mtHsp70	mitochondrial heat shock protein
mTOR	mammalian target of rapamycin
nAMD	neovascular age-related macular degeneration
NRLP3	NLR Family Pyrin Domain Containing 3
PAI-1	plasminogen activator inhibitor type-1
PECAM-1	platelet-endothelial cell adhesion molecule-1
PET-MRI	positron emission tomography-magnetic resonance imaging
PDGF	platelet-derived growth factor
PDR	proliferative diabetic retinopathy
PGC1α	proliferator-activated receptor gamma coactivator 1-alpha
PI3K	phosphatidylinositol-3 kinase
PVR	proliferative vitreoretinopathy
OIR	oxygen-induced retinopathy
OxLDL	oxidized low density lipoprotein
Rho GTPase	ras-homologous GTPase
ROP	retinopathy of prematurity
ROS	reactive oxygen species
RPE	retinal pigment epithelium
RTK	receptor tyrosine kinase
RUNX1	runt-related transcription factor 1
siRNA	small interfering RNA
S1P	sphingosine-1-phosphate
Taz	transcriptional co-activator with PDZ-binding motif
TCF/LEF	t-cell factor/lymphoid enhancer-binding factor
Tead	transcriptional enhanced associate domains
TFs	transcription factors
TGFβ	transforming growth factor-β
TGFBR1	transforming growth factor-β receptor 1
TIE	tyrosine kinase with immunoglobulin-like and EGF-like domains 1
TIMPs	tissue inhibitors of matrix metalloproteinase
TNF-α	tumor necrosis factor-α
TRPV4	transient receptor potential vanilloid 4
TSP1	thrombospondin 1
uPA	urokinase-type plasminogen activator
uPAR	urokinase-type plasminogen activator receptor
VCAM	vascular cell adhesion molecule
VE-Cadherin	vascular endothelial-cadherin
VEGF	vascular endothelial growth factor
vWF	von Willebrand Factor
Wnt	Wingless/Integrated
YAP	Yes-associated protein
Zeb	zinc finger E-box-binding homeobox
ZO-1	zonula occludens-1

## References

[1] Ishikawa K., Kannan R., Hinton D.R. (2016). Molecular mechanisms of subretinal fibrosis in age-related macular degeneration. Exp. Eye Res..

[2] Gabbiani G. (2003). The myofibroblast in wound healing and fibrocontractive diseases. J. Pathol..

[3] Shu D.Y., Lovicu F.J. (2017). Myofibroblast transdifferentiation: The dark force in ocular wound healing and fibrosis. Prog. Retin. Eye Res..

[4] Radeke M.J., Radeke C.M., Shih Y.H., Hu J., Bok D., Johnson L.V., Coffey P.J. (2015). Restoration of mesenchymal retinal pigmented epithelial cells by TGFbeta pathway inhibitors: Implications for age-related macular degeneration. Genome Med..

[5] Sun J.X., Chang T.F., Li M.H., Sun L.J., Yan X.C., Yang Z.Y., Liu Y., Xu W.Q., Lv Y., Su J.B. (2018). SNAI1, an endothelial-mesenchymal transition transcription factor, promotes the early phase of ocular neovascularization. Angiogenesis.

[6] Siemerink M.J., Augustin A.J., Schlingemann R.O. (2010). Mechanisms of ocular angiogenesis and its molecular mediators. Dev. Ophthalmol..

[7] Tamiya S., Kaplan H.J. (2016). Role of epithelial-mesenchymal transition in proliferative vitreoretinopathy. Exp. Eye Res..

[8] Boles N.C., Fernandes M., Swigut T., Srinivasan R., Schiff L., Rada-Iglesias A., Wang Q., Saini J.S., Kiehl T., Stern J.H. (2020). Epigenomic and Transcriptomic Changes During Human RPE EMT in a Stem Cell Model of Epiretinal Membrane Pathogenesis and Prevention by Nicotinamide. Stem Cell Rep..

[9] Friedlander M. (2007). Fibrosis and diseases of the eye. J. Clin. Investig..

[10] Abu El-Asrar A.M., De Hertogh G., van den Eynde K., Alam K., Van Raemdonck K., Opdenakker G., Van Damme J., Geboes K., Struyf S. (2015). Myofibroblasts in proliferative diabetic retinopathy can originate from infiltrating fibrocytes and through endothelial-to-mesenchymal transition (EndoMT). Exp. Eye Res..

[11] Stone R.C., Pastar I., Ojeh N., Chen V., Liu S., Garzon K.I., Tomic-Canic M. (2016). Epithelial-mesenchymal transition in tissue repair and fibrosis. Cell Tissue Res..

[12] Kalluri R., Weinberg R.A. (2009). The basics of epithelial-mesenchymal transition. J. Clin Investig..

[13] Kovacic J.C., Mercader N., Torres M., Boehm M., Fuster V. (2012). Epithelial-to-mesenchymal and endothelial-to-mesenchymal transition: From cardiovascular development to disease. Circulation.

[14] Saito A. (2013). EMT and EndMT: Regulated in similar ways?. J. Biochem..

[15] Medici D., Kalluri R. (2012). Endothelial-mesenchymal transition and its contribution to the emergence of stem cell phenotype. Semin. Cancer Biol..

[16] Neve A., Cantatore F.P., Maruotti N., Corrado A., Ribatti D. (2014). Extracellular matrix modulates angiogenesis in physiological and pathological conditions. Biomed. Res. Int..

[17] Scott L.E., Weinberg S.H., Lemmon C.A. (2019). Mechanochemical Signaling of the Extracellular Matrix in Epithelial-Mesenchymal Transition. Front. Cell Dev. Biol..

[18] Welch-Reardon K.M., Wu N., Hughes C.C. (2015). A role for partial endothelial-mesenchymal transitions in angiogenesis?. Arterioscler. Thromb. Vasc. Biol..

[19] Saitoh M. (2018). Involvement of partial EMT in cancer progression. J. Biochem..

[20] Park J.A., Kim J.H., Bi D., Mitchel J.A., Qazvini N.T., Tantisira K., Park C.Y., McGill M., Kim S.H., Gweon B. (2015). Unjamming and cell shape in the asthmatic airway epithelium. Nat. Mater..

[21] Park J.A., Atia L., Mitchel J.A., Fredberg J.J., Butler J.P. (2016). Collective migration and cell jamming in asthma, cancer and development. J. Cell Sci..

[22] Pérez L., Muñoz-Durango N., Riedel C.A., Echeverría C., Kalergis A.M., Cabello-Verrugio C., Simon F. (2017). Endothelial-to-mesenchymal transition: Cytokine-mediated pathways that determine endothelial fibrosis under inflammatory conditions. Cytokine Growth Factor Rev..

[23] Nieto M.A., Huang R.Y., Jackson R.A., Thiery J.P. (2016). Emt: 2016. Cell.

[24] Saika S. (2006). TGFbeta pathobiology in the eye. Lab. Investig..

[25] Annes J.P., Munger J.S., Rifkin D.B. (2003). Making sense of latent TGFbeta activation. J. Cell Sci..

[26] Horiguchi M., Ota M., Rifkin D.B. (2012). Matrix control of transforming growth factor-beta function. J. Biochem..

[27] Robertson I.B., Rifkin D.B. (2016). Regulation of the Bioavailability of TGF-beta and TGF-beta-Related Proteins. Cold Spring Harb. Perspect. Biol..

[28] Luo K. (2017). Signaling Cross Talk between TGF-β/Smad and Other Signaling Pathways. Cold Spring Harb. Perspect. Biol..

[29] Connor T.B., Roberts A.B., Sporn M.B., Danielpour D., Dart L.L., Michels R.G., de Bustros S., Enger C., Kato H., Lansing M. (1989). Correlation of fibrosis and transforming growth factor-beta type 2 levels in the eye. J. Clin Investig..

[30] Tosi G.M., Orlandini M., Galvagni F. (2018). The Controversial Role of TGF-β in Neovascular Age-Related Macular Degeneration Pathogenesis. Int. J. Mol. Sci..

[31] Tosi G.M., Neri G., Caldi E., Fusco F., Bacci T., Tarantello A., Nuti E., Marigliani D., Baiocchi S., Traversi C. (2018). TGF-beta concentrations and activity are down-regulated in the aqueous humor of patients with neovascular age-related macular degeneration. Sci. Rep..

[32] Saika S., Saika S., Liu C.Y., Azhar M., Sanford L.P., Doetschman T., Gendron R.L., Kao C.W., Kao W.W. (2001). TGFbeta2 in corneal morphogenesis during mouse embryonic development. Dev. Biol..

[33] Sanford L.P., Ormsby I., Gittenberger-de Groot A.C., Sariola H., Friedman R., Boivin G.P., Cardell E.L., Doetschman T. (1997). TGFβ2 knockout mice have multiple developmental defects that are non-overlapping with other TGFβ knockout phenotypes. Development.

[34] Pfeffer B.A., Flanders K.C., Guérin C.J., Danielpour D., Anderson D.H. (1994). Transforming growth factor beta 2 is the predominant isoform in the neural retina, retinal pigment epithelium-choroid and vitreous of the monkey eye. Exp. Eye Res..

[35] Tanihara H., Yoshida M., Matsumoto M., Yoshimura N. (1993). Identification of transforming growth factor-beta expressed in cultured human retinal pigment epithelial cells. Investig. Ophthalmol. Vis. Sci..

[36] Kvanta A. (1994). Expression and secretion of transforming growth factor-beta in transformed and nontransformed retinal pigment epithelial cells. Ophthalmic Res..

[37] Hirsch L., Nazari H., Sreekumar P.G., Kannan R., Dustin L., Zhu D., Barron E., Hinton D.R. (2015). TGF-beta2 secretion from RPE decreases with polarization and becomes apically oriented. Cytokine.

[38] Mochizuki M., Sugita S., Kamoi K. (2013). Immunological homeostasis of the eye. Prog. Retin. Eye Res..

[39] Grossniklaus H.E., Green W.R. (1998). Histopathologic and ultrastructural findings of surgically excised choroidal neovascularization. Submacular Surgery Trials Research Group. Arch. Ophthalmol..

[40] Lopez P.F., Sippy B.D., Lambert H.M., Thach A.B., Hinton D.R. (1996). Transdifferentiated retinal pigment epithelial cells are immunoreactive for vascular endothelial growth factor in surgically excised age-related macular degeneration-related choroidal neovascular membranes. Investig. Ophthalmol. Vis. Sci..

[41] Grossniklaus H.E., Hutchinson A.K., Capone A., Woolfson J., Lambert H.M. (1994). Clinicopathologic features of surgically excised choroidal neovascular membranes. Ophthalmology.

[42] Sarks J.P., Sarks S.H., Killingsworth M.C. (1988). Evolution of geographic atrophy of the retinal pigment epithelium. Eye.

[43] Guidry C., Medeiros N.E., Curcio C.A. (2002). Phenotypic variation of retinal pigment epithelium in age-related macular degeneration. Investig. Ophthalmol. Vis. Sci..

[44] Zanzottera E.C., Messinger J.D., Ach T., Smith R.T., Curcio C.A. (2015). Subducted and melanotic cells in advanced age-related macular degeneration are derived from retinal pigment epithelium. Investig. Ophthalmol. Vis. Sci..

[45] Kaufhold S., Bonavida B. (2014). Central role of Snail1 in the regulation of EMT and resistance in cancer: A target for therapeutic intervention. J. Exp. Clin. Cancer Res..

[46] Kokudo T., Suzuki Y., Yoshimatsu Y., Yamazaki T., Watabe T., Miyazono K. (2008). Snail is required for TGFbeta-induced endothelial-mesenchymal transition of embryonic stem cell-derived endothelial cells. J. Cell Sci..

[47] Lopez D., Niu G., Huber P., Carter W.B. (2009). Tumor-induced upregulation of Twist, Snail, and Slug represses the activity of the human VE-cadherin promoter. Arch. Biochem. Biophys..

[48] Ghosh S., Shang P., Terasaki H., Stepicheva N., Hose S., Yazdankhah M., Weiss J., Sakamoto T., Bhutto I.A., Xia S. (2018). A Role for betaA3/A1-Crystallin in Type 2 EMT of RPE Cells Occurring in Dry Age-Related Macular Degeneration. Investig. Ophthalmol Vis. Sci..

[49] Hirasawa M., Noda K., Noda S., Suzuki M., Ozawa Y., Shinoda K., Inoue M., Ogawa Y., Tsubota K., Ishida S. (2011). Transcriptional factors associated with epithelial-mesenchymal transition in choroidal neovascularization. Mol. Vis..

[50] Feng Z., Li R., Shi H., Bi W., Hou W., Zhang X. (2015). Combined silencing of TGF-beta2 and Snail genes inhibit epithelial-mesenchymal transition of retinal pigment epithelial cells under hypoxia. Graefes Arch. Clin. Exp. Ophthalmol..

[51] Saika S., Kono-Saika S., Tanaka T., Yamanaka O., Ohnishi Y., Sato M., Muragaki Y., Ooshima A., Yoo J., Flanders K.C. (2004). Smad3 is required for dedifferentiation of retinal pigment epithelium following retinal detachment in mice. Lab. Investig..

[52] Tamiya S., Liu L., Kaplan H.J. (2010). Epithelial-mesenchymal transition and proliferation of retinal pigment epithelial cells initiated upon loss of cell-cell contact. Investig. Ophthalmol. Vis. Sci..

[53] Piera-Velazquez S., Jimenez S.A. (2019). Endothelial to Mesenchymal Transition: Role in Physiology and in the Pathogenesis of Human Diseases. Physiol. Rev..

[54] Niessen C.M. (2007). Tight junctions/adherens junctions: Basic structure and function. J. Investig. Dermatol.

[55] Lampugnani M.G. (2012). Endothelial cell-to-cell junctions: Adhesion and signaling in physiology and pathology. Cold Spring Harb. Perspect. Med..

[56] Nelson W.J., Nusse R. (2004). Convergence of Wnt, beta-catenin, and cadherin pathways. Science.

[57] Liebner S., Cattelino A., Gallini R., Rudini N., Iurlaro M., Piccolo S., Dejana E. (2004). Beta-catenin is required for endothelial-mesenchymal transformation during heart cushion development in the mouse. J. Cell Biol..

[58] Mylavarapu S., Kumar H., Kumari S., Sravanthi L.S., Jain M., Basu A., Biswas M., Mylavarapu S.V.S., Das A., Roy M. (2019). Activation of Epithelial-Mesenchymal Transition and Altered beta-Catenin Signaling in a Novel Indian Colorectal Carcinoma Cell Line. Front. Oncol..

[59] Pratt C.H., Vadigepalli R., Chakravarthula P., Gonye G.E., Philp N.J., Grunwald G.B. (2008). Transcriptional regulatory network analysis during epithelial-mesenchymal transformation of retinal pigment epithelium. Mol. Vis..

[60] Chen H.C., Zhu Y.T., Chen S.Y., Tseng S.C. (2012). Wnt signaling induces epithelial-mesenchymal transition with proliferation in ARPE-19 cells upon loss of contact inhibition. Lab. Investig..

[61] Umazume K., Tsukahara R., Liu L., Fernandez de Castro J.P., McDonald K., Kaplan H.J., Tamiya S. (2014). Role of retinal pigment epithelial cell beta-catenin signaling in experimental proliferative vitreoretinopathy. Am. J. Pathol..

[62] Kim M., Kim T., Johnson R.L., Lim D.S. (2015). Transcriptional co-repressor function of the hippo pathway transducers YAP and TAZ. Cell Rep..

[63] Lilien J., Balsamo J. (2005). The regulation of cadherin-mediated adhesion by tyrosine phosphorylation/dephosphorylation of beta-catenin. Curr. Opin. Cell Biol..

[64] Umazume K., Liu L., Scott P.A., de Castro J.P., McDonald K., Kaplan H.J., Tamiya S. (2013). Inhibition of PVR with a tyrosine kinase inhibitor, dasatinib, in the swine. Investig. Ophthalmol. Vis. Sci..

[65] Liu Y., Xin Y., Ye F., Wang W., Lu Q., Kaplan H.J., Dean D.C. (2010). Taz-tead1 links cell-cell contact to zeb1 expression, proliferation, and dedifferentiation in retinal pigment epithelial cells. Investig. Ophthalmol. Vis. Sci..

[66] Chen X., Xiao W., Liu X., Zeng M., Luo L., Wu M., Ye S., Liu Y. (2014). Blockade of Jagged/Notch pathway abrogates transforming growth factor beta2-induced epithelial-mesenchymal transition in human retinal pigment epithelium cells. Curr. Mol. Med..

[67] Chen X., Xiao W., Wang W., Luo L., Ye S., Liu Y. (2014). The complex interplay between ERK1/2, TGFβ/Smad, and Jagged/Notch signaling pathways in the regulation of epithelial-mesenchymal transition in retinal pigment epithelium cells. PLoS ONE.

[68] Kimoto K., Nakatsuka K., Matsuo N., Yoshioka H. (2004). p38 MAPK mediates the expression of type I collagen induced by TGF-beta 2 in human retinal pigment epithelial cells ARPE-19. Investig. Ophthalmol. Vis. Sci..

[69] Schiff L., Boles N.C., Fernandes M., Nachmani B., Gentile R., Blenkinsop T.A. (2019). P38 inhibition reverses TGFbeta1 and TNFalpha-induced contraction in a model of proliferative vitreoretinopathy. Commun. Biol..

[70] Fritsche L.G., Chen W., Schu M., Yaspan B.L., Yu Y., Thorleifsson G., Zack D.J., Arakawa S., Cipriani V., Ripke S. (2013). Seven new loci associated with age-related macular degeneration. Nat. Genet..

[71] Zarranz-Ventura J., Fernandez-Robredo P., Recalde S., Salinas-Alaman A., Borras-Cuesta F., Dotor J., Garcia-Layana A. (2013). Transforming growth factor-beta inhibition reduces progression of early choroidal neovascularization lesions in rats: P17 and P144 peptides. PLoS ONE.

[72] Recalde S., Zarranz-Ventura J., Fernandez-Robredo P., Garcia-Gomez P.J., Salinas-Alaman A., Borras-Cuesta F., Dotor J., Garcia-Layana A. (2011). Transforming growth factor-beta inhibition decreases diode laser-induced choroidal neovascularization development in rats: P17 and P144 peptides. Investig. Ophthalmol. Vis. Sci..

[73] Zhang H., Liu Z.L. (2012). Transforming growth factor-beta neutralizing antibodies inhibit subretinal fibrosis in a mouse model. Int. J. Ophthalmol..

[74] Bai Y., Liang S., Yu W., Zhao M., Huang L., Zhao M., Li X. (2014). Semaphorin 3A blocks the formation of pathologic choroidal neovascularization induced by transforming growth factor beta. Mol. Vis..

[75] Lebrun J.J. (2012). The Dual Role of TGFbeta in Human Cancer: From Tumor Suppression to Cancer Metastasis. ISRN Mol. Biol..

[76] Klaassen I., van Geest R.J., Kuiper E.J., van Noorden C.J., Schlingemann R.O. (2015). The role of CTGF in diabetic retinopathy. Exp. Eye Res..

[77] Kuiper E.J., Van Nieuwenhoven F.A., de Smet M.D., van Meurs J.C., Tanck M.W., Oliver N., Klaassen I., Van Noorden C.J., Goldschmeding R., Schlingemann R.O. (2008). The angio-fibrotic switch of VEGF and CTGF in proliferative diabetic retinopathy. PLoS ONE.

[78] Cicha I., Goppelt-Struebe M. (2009). Connective tissue growth factor: Context-dependent functions and mechanisms of regulation. Biofactors.

[79] Inoki I., Shiomi T., Hashimoto G., Enomoto H., Nakamura H., Makino K., Ikeda E., Takata S., Kobayashi K., Okada Y. (2002). Connective tissue growth factor binds vascular endothelial growth factor (VEGF) and inhibits VEGF-induced angiogenesis. FASEB J..

[80] Hu B., Zhang Y., Zeng Q., Han Q., Zhang L., Liu M., Li X. (2014). Intravitreal injection of ranibizumab and CTGF shRNA improves retinal gene expression and microvessel ultrastructure in a rodent model of diabetes. Int. J. Mol. Sci..

[81] Johnson L.V., Ozaki S., Staples M.K., Erickson P.A., Anderson D.H. (2000). A potential role for immune complex pathogenesis in drusen formation. Exp. Eye Res..

[82] Mullins R.F., Russell S.R., Anderson D.H., Hageman G.S. (2000). Drusen associated with aging and age-related macular degeneration contain proteins common to extracellular deposits associated with atherosclerosis, elastosis, amyloidosis, and dense deposit disease. FASEB J..

[83] Tseng W.A., Thein T., Kinnunen K., Lashkari K., Gregory M.S., D’Amore P.A., Ksander B.R. (2013). NLRP3 inflammasome activation in retinal pigment epithelial cells by lysosomal destabilization: Implications for age-related macular degeneration. Investig. Ophthalmol. Vis. Sci..

[84] Ambati J., Atkinson J.P., Gelfand B.D. (2013). Immunology of age-related macular degeneration. Nat. Rev. Immunol..

[85] Cui W., Zhang H., Liu Z.L. (2014). Interleukin-6 receptor blockade suppresses subretinal fibrosis in a mouse model. Int. J. Ophthalmol..

[86] Jo Y.J., Sonoda K.H., Oshima Y., Takeda A., Kohno R., Yamada J., Hamuro J., Yang Y., Notomi S., Hisatomi T. (2011). Establishment of a new animal model of focal subretinal fibrosis that resembles disciform lesion in advanced age-related macular degeneration. Investig. Ophthalmol. Vis. Sci..

[87] Cui L., Lyu Y., Jin X., Wang Y., Li X., Wang J., Zhang J., Deng Z., Yang N., Zheng Z. (2019). miR-194 suppresses epithelial-mesenchymal transition of retinal pigment epithelial cells by directly targeting ZEB1. Ann. Transl. Med..

[88] Zhang R., Liu Z., Zhang H., Zhang Y., Lin D. (2016). The COX-2-Selective Antagonist (NS-398) Inhibits Choroidal Neovascularization and Subretinal Fibrosis. PLoS ONE.

[89] Ishikawa K., He S., Terasaki H., Nazari H., Zhang H., Spee C., Kannan R., Hinton D.R. (2015). Resveratrol inhibits epithelial-mesenchymal transition of retinal pigment epithelium and development of proliferative vitreoretinopathy. Sci. Rep..

[90] Krstic J., Trivanovic D., Mojsilovic S., Santibanez J.F. (2015). Transforming Growth Factor-Beta and Oxidative Stress Interplay: Implications in Tumorigenesis and Cancer Progression. Oxidative Med. Cell Longev..

[91] Li G., Li C.X., Xia M., Ritter J.K., Gehr T.W.B., Boini K., Li P.L. (2015). Enhanced Epithelial-to-Mesenchymal Transition Associated with Lysosome Dysfunction in Podocytes: Role of p62/Sequestosome 1 as a Signaling Hub. Cell. Physiol. Biochem..

[92] Zhao Z., Zhao J., Xue J., Zhao X., Liu P. (2016). Autophagy inhibition promotes epithelial-mesenchymal transition through ROS/HO-1 pathway in ovarian cancer cells. Am. J. Cancer Res..

[93] Stefansson E., Geirsdottir A., Sigurdsson H. (2011). Metabolic physiology in age related macular degeneration. Prog. Retin. Eye Res..

[94] Kaarniranta K., Uusitalo H., Blasiak J., Felszeghy S., Kannan R., Kauppinen A., Salminen A., Sinha D., Ferrington D. (2020). Mechanisms of mitochondrial dysfunction and their impact on age-related macular degeneration. Prog. Retin. Eye Res..

[95] Feher J., Kovacs I., Artico M., Cavallotti C., Papale A., Balacco Gabrieli C. (2006). Mitochondrial alterations of retinal pigment epithelium in age-related macular degeneration. Neurobiol. Aging.

[96] Terluk M.R., Kapphahn R.J., Soukup L.M., Gong H., Gallardo C., Montezuma S.R., Ferrington D.A. (2015). Investigating mitochondria as a target for treating age-related macular degeneration. J. Neurosci..

[97] Karunadharma P.P., Nordgaard C.L., Olsen T.W., Ferrington D.A. (2010). Mitochondrial DNA damage as a potential mechanism for age-related macular degeneration. Investig. Ophthalmol. Vis. Sci..

[98] Udar N., Atilano S.R., Memarzadeh M., Boyer D.S., Chwa M., Lu S., Maguen B., Langberg J., Coskun P., Wallace D.C. (2009). Mitochondrial DNA haplogroups associated with age-related macular degeneration. Investig. Ophthalmol. Vis. Sci..

[99] Kenney M.C., Atilano S.R., Boyer D., Chwa M., Chak G., Chinichian S., Coskun P., Wallace D.C., Nesburn A.B., Udar N.S. (2010). Characterization of retinal and blood mitochondrial DNA from age-related macular degeneration patients. Investig. Ophthalmol. Vis. Sci..

[100] Nordgaard C.L., Karunadharma P.P., Feng X., Olsen T.W., Ferrington D.A. (2008). Mitochondrial proteomics of the retinal pigment epithelium at progressive stages of age-related macular degeneration. Investig. Ophthalmol. Vis. Sci..

[101] Nordgaard C.L., Berg K.M., Kapphahn R.J., Reilly C., Feng X., Olsen T.W., Ferrington D.A. (2006). Proteomics of the retinal pigment epithelium reveals altered protein expression at progressive stages of age-related macular degeneration. Investig. Ophthalmol. Vis. Sci..

[102] Ferrington D.A., Ebeling M.C., Kapphahn R.J., Terluk M.R., Fisher C.R., Polanco J.R., Roehrich H., Leary M.M., Geng Z., Dutton J.R. (2017). Altered bioenergetics and enhanced resistance to oxidative stress in human retinal pigment epithelial cells from donors with age-related macular degeneration. Redox Biol..

[103] Golestaneh N., Chu Y., Xiao Y.Y., Stoleru G.L., Theos A.C. (2017). Dysfunctional autophagy in RPE, a contributing factor in age-related macular degeneration. Cell Death Dis..

[104] Matoba R., Morizane Y., Shiode Y., Hirano M., Doi S., Toshima S., Araki R., Hosogi M., Yonezawa T., Shiraga F. (2017). Suppressive effect of AMP-activated protein kinase on the epithelial-mesenchymal transition in retinal pigment epithelial cells. PLoS ONE.

[105] Shukal D., Bhadresha K., Shastri B., Mehta D., Vasavada A., Johar K. (2020). Dichloroacetate prevents TGFβ-induced epithelial-mesenchymal transition of retinal pigment epithelial cells. Exp. Eye Res..

[106] Che D., Zhou T., Lan Y., Xie J., Gong H., Li C., Feng J., Hong H., Qi W., Ma C. (2016). High glucose-induced epithelial-mesenchymal transition contributes to the upregulation of fibrogenic factors in retinal pigment epithelial cells. Int. J. Mol. Med..

[107] Cao Y., Feng B., Chen S., Chu Y., Chakrabarti S. (2014). Mechanisms of endothelial to mesenchymal transition in the retina in diabetes. Investig. Ophthalmol. Vis. Sci..

[108] Kaarniranta K., Kajdanek J., Morawiec J., Pawlowska E., Blasiak J. (2018). PGC-1α Protects RPE Cells of the Aging Retina against Oxidative Stress-Induced Degeneration through the Regulation of Senescence and Mitochondrial Quality Control. The Significance for AMD Pathogenesis. Int. J. Mol. Sci..

[109] Zhang M., Chu Y., Mowery J., Konkel B., Galli S., Theos A.C., Golestaneh N. (2018). Pgc-1α repression and high-fat diet induce age-related macular degeneration-like phenotypes in mice. Dis. Models Mech..

[110] Iacovelli J., Rowe G.C., Khadka A., Diaz-Aguilar D., Spencer C., Arany Z., Saint-Geniez M. (2016). PGC-1α Induces Human RPE Oxidative Metabolism and Antioxidant Capacity. Investig. Ophthalmol. Vis. Sci..

[111] Rosales M.A.B., Shu D.Y., Iacovelli J., Saint-Geniez M. (2019). Loss of PGC-1alpha in RPE induces mesenchymal transition and promotes retinal degeneration. Life Sci. Alliance.

[112] Satish S., Philipose H., Rosales M.A.B., Saint-Geniez M. (2018). Pharmaceutical Induction of PGC-1alpha Promotes Retinal Pigment Epithelial Cell Metabolism and Protects against Oxidative Damage. Oxidative Med. Cell Longev..

[113] Sinha D., Valapala M., Shang P., Hose S., Grebe R., Lutty G.A., Zigler J.S., Kaarniranta K., Handa J.T. (2016). Lysosomes: Regulators of autophagy in the retinal pigmented epithelium. Exp. Eye Res..

[114] Koh J.Y., Kim H.N., Hwang J.J., Kim Y.H., Park S.E. (2019). Lysosomal dysfunction in proteinopathic neurodegenerative disorders: Possible therapeutic roles of cAMP and zinc. Mol. Brain.

[115] Kaarniranta K., Sinha D., Blasiak J., Kauppinen A., Vereb Z., Salminen A., Boulton M.E., Petrovski G. (2013). Autophagy and heterophagy dysregulation leads to retinal pigment epithelium dysfunction and development of age-related macular degeneration. Autophagy.

[116] Hyttinen J.M.T., Viiri J., Kaarniranta K., Błasiak J. (2018). Mitochondrial quality control in AMD: Does mitophagy play a pivotal role?. Cell Mol. Life Sci..

[117] Guerra F., Guaragnella N., Arbini A.A., Bucci C., Giannattasio S., Moro L. (2017). Mitochondrial Dysfunction: A Novel Potential Driver of Epithelial-to-Mesenchymal Transition in Cancer. Front. Oncol..

[118] Zou J., Liu Y., Li B., Zheng Z., Ke X., Hao Y., Li X., Li X., Liu F., Zhang Z. (2017). Autophagy attenuates endothelial-to-mesenchymal transition by promoting Snail degradation in human cardiac microvascular endothelial cells. Biosci. Rep..

[119] Ishikawa M., Sawada Y., Yoshitomi T. (2015). Structure and function of the interphotoreceptor matrix surrounding retinal photoreceptor cells. Exp. Eye Res..

[120] Pauleikhoff D., Harper C.A., Marshall J., Bird A.C. (1990). Aging changes in Bruch’s membrane. A histochemical and morphologic study. Ophthalmology.

[121] Marshall G.E., Konstas A.G., Reid G.G., Edwards J.G., Lee W.R. (1992). Type IV collagen and laminin in Bruch’s membrane and basal linear deposit in the human macula. Br. J. Ophthalmol..

[122] Curcio C.A., Johnson M. (2012). Structure, Function, and Pathology of Bruch’s Membrane. Retina.

[123] Sun K., Cai H., Tezel T.H., Paik D., Gaillard E.R., Del Priore L.V. (2007). Bruch’s membrane aging decreases phagocytosis of outer segments by retinal pigment epithelium. Mol. Vis..

[124] Ramrattan R.S., van der Schaft T.L., Mooy C.M., de Bruijn W.C., Mulder P.G., de Jong P.T. (1994). Morphometric analysis of Bruch’s membrane, the choriocapillaris, and the choroid in aging. Investig. Ophthalmol. Vis. Sci..

[125] Moore D.J., Hussain A.A., Marshall J. (1995). Age-related variation in the hydraulic conductivity of Bruch’s membrane. Investig. Ophthalmol. Vis. Sci..

[126] Del Priore L.V., Tezel T.H. (1998). Reattachment rate of human retinal pigment epithelium to layers of human Bruch’s membrane. Arch. Ophthalmol..

[127] Cai H., Del Priore L.V. (2006). Bruch membrane aging alters the gene expression profile of human retinal pigment epithelium. Curr. Eye Res..

[128] Gullapalli V.K., Sugino I.K., Van Patten Y., Shah S., Zarbin M.A. (2005). Impaired RPE survival on aged submacular human Bruch’s membrane. Exp. Eye Res..

[129] Wang H., Ninomiya Y., Sugino I.K., Zarbin M.A. (2003). Retinal pigment epithelium wound healing in human Bruch’s membrane explants. Investig. Ophthalmol. Vis. Sci..

[130] Tezel T.H., Kaplan H.J., Del Priore L.V. (1999). Fate of human retinal pigment epithelial cells seeded onto layers of human Bruch’s membrane. Investig. Ophthalmol. Vis. Sci..

[131] Thomas M.A., Dickinson J.D., Melberg N.S., Ibanez H.E., Dhaliwal R.S. (1994). Visual results after surgical removal of subfoveal choroidal neovascular membranes. Ophthalmology.

[132] Heller J.P., Martin K.R. (2014). Enhancing RPE Cell-Based Therapy Outcomes for AMD: The Role of Bruch’s Membrane. Transl. Vis. Sci. Technol..

[133] Nita M., Strzalka-Mrozik B., Grzybowski A., Mazurek U., Romaniuk W. (2014). Age-related macular degeneration and changes in the extracellular matrix. Med. Sci. Monit..

[134] Das A., Puklin J.E., Frank R.N., Zhang N.L. (1992). Ultrastructural immunocytochemistry of subretinal neovascular membranes in age-related macular degeneration. Ophthalmology.

[135] Chau K.Y., Sivaprasad S., Patel N., Donaldson T.A., Luthert P.J., Chong N.V. (2007). Plasma levels of matrix metalloproteinase-2 and -9 (MMP-2 and MMP-9) in age-related macular degeneration. Eye (Lond.).

[136] Marin-Castano M.E., Csaky K.G., Cousins S.W. (2005). Nonlethal oxidant injury to human retinal pigment epithelium cells causes cell membrane blebbing but decreased MMP-2 activity. Investig. Ophthalmol. Vis. Sci..

[137] Marin-Castano M.E., Striker G.E., Alcazar O., Catanuto P., Espinosa-Heidmann D.G., Cousins S.W. (2006). Repetitive nonlethal oxidant injury to retinal pigment epithelium decreased extracellular matrix turnover in vitro and induced sub-RPE deposits in vivo. Investig. Ophthalmol. Vis. Sci..

[138] Steen B., Sejersen S., Berglin L., Seregard S., Kvanta A. (1998). Matrix metalloproteinases and metalloproteinase inhibitors in choroidal neovascular membranes. Investig. Ophthalmol. Vis. Sci..

[139] Plantner J.J., Jiang C., Smine A. (1998). Increase in interphotoreceptor matrix gelatinase A (MMP-2) associated with age-related macular degeneration. Exp. Eye Res..

[140] Behzadian M.A., Wang X.L., Windsor L.J., Ghaly N., Caldwell R.B. (2001). TGF-beta increases retinal endothelial cell permeability by increasing MMP-9: Possible role of glial cells in endothelial barrier function. Investig. Ophthalmol. Vis. Sci..

[141] Elner S.G., Elner V.M., Kindzelskii A.L., Horino K., Davis H.R., Todd R.F., Glagov S., Petty H.R. (2003). Human RPE cell lysis of extracellular matrix: Functional urokinase plasminogen activator receptor (uPAR), collagenase and elastase. Exp. Eye Res..

[142] Siren V., Myohanen H., Vaheri A., Immonen I. (1999). Transforming growth factor beta induces urokinase receptor expression in cultured retinal pigment epithelial cells. Ophthalmic Res..

[143] Sugioka K., Kodama A., Okada K., Iwata M., Yoshida K., Kusaka S., Matsumoto C., Kaji H., Shimomura Y. (2013). TGF-β2 promotes RPE cell invasion into a collagen gel by mediating urokinase-type plasminogen activator (uPA) expression. Exp. Eye Res..

[144] Ozkaya A., Erdogan G., Tarakcioglu H.N. (2018). Submacular hemorrhage secondary to age-related macular degeneration managed with vitrectomy, subretinal injection of tissue plasminogen activator, hemorrhage displacement with liquid perfluorocarbon, gas tamponade, and face-down positioning. Saudi J. Ophthalmol..

[145] Lambert V., Munaut C., Carmeliet P., Gerard R.D., Declerck P.J., Gils A., Claes C., Foidart J.M., Noel A., Rakic J.M. (2003). Dose-dependent modulation of choroidal neovascularization by plasminogen activator inhibitor type I: Implications for clinical trials. Investig. Ophthalmol. Vis. Sci..

[146] Pepper M.S., Sappino A.P., Montesano R., Orci L., Vassalli J.D. (1992). Plasminogen activator inhibitor-1 is induced in migrating endothelial cells. J. Cell Physiol..

[147] Basu A., Menicucci G., Maestas J., Das A., McGuire P. (2009). Plasminogen activator inhibitor-1 (PAI-1) facilitates retinal angiogenesis in a model of oxygen-induced retinopathy. Investig. Ophthalmol. Vis. Sci..

[148] Swaney J.S., Moreno K.M., Gentile A.M., Sabbadini R.A., Stoller G.L. (2008). Sphingosine-1-phosphate (S1P) is a novel fibrotic mediator in the eye. Exp. Eye Res..

[149] Penn J.S., Rajaratnam V.S. (2003). Inhibition of retinal neovascularization by intravitreal injection of human rPAI-1 in a rat model of retinopathy of prematurity. Investig. Ophthalmol. Vis. Sci..

[150] Lambert V., Munaut C., Noel A., Frankenne F., Bajou K., Gerard R., Carmeliet P., Defresne M.P., Foidart J.M., Rakic J.M. (2001). Influence of plasminogen activator inhibitor type 1 on choroidal neovascularization. FASEB J..

[151] Tschumperlin D.J., Lagares D. (2020). Mechano-therapeutics: Targeting Mechanical Signaling in Fibrosis and Tumor Stroma. Pharmacol. Ther..

[152] Martino F., Perestrelo A.R., Vinarsky V., Pagliari S., Forte G. (2018). Cellular Mechanotransduction: From Tension to Function. Front. Physiol..

[153] Kechagia J.Z., Ivaska J., Roca-Cusachs P. (2019). Integrins as biomechanical sensors of the microenvironment. Nat. Rev. Mol. Cell Biol..

[154] Cui J., Maberley D., Samad A., Ma P., Ning A., Matsubara J.A., Baciu P. (2009). Expression of integrins on human choroidal neovascular membranes. J. Ocul. Biol. Dis. Inform..

[155] Hou X., Han Q.H., Hu D., Tian L., Guo C.M., Du H.J., Zhang P., Wang Y.S., Hui Y.N. (2009). Mechanical force enhances MMP-2 activation via p38 signaling pathway in human retinal pigment epithelial cells. Graefes Arch. Clin. Exp. Ophthalmol..

[156] Sharma S., Goswami R., Zhang D.X., Rahaman S.O. (2019). TRPV4 regulates matrix stiffness and TGFbeta1-induced epithelial-mesenchymal transition. J. Cell Mol. Med..

[157] Lee W.H., Choong L.Y., Mon N.N., Lu S., Lin Q., Pang B., Yan B., Krishna V.S., Singh H., Tan T.Z. (2016). TRPV4 Regulates Breast Cancer Cell Extravasation, Stiffness and Actin Cortex. Sci. Rep..

[158] Okada Y., Shirai K., Miyajima M., Reinach P.S., Yamanaka O., Sumioka T., Kokado M., Tomoyose K., Saika S. (2016). Loss of TRPV4 Function Suppresses Inflammatory Fibrosis Induced by Alkali-Burning Mouse Corneas. PLoS ONE.

[159] Arredondo Zamarripa D., Noguez Imm R., Bautista Cortes A.M., Vazquez Ruiz O., Bernardini M., Fiorio Pla A., Gkika D., Prevarskaya N., Lopez-Casillas F., Liedtke W. (2017). Dual contribution of TRPV4 antagonism in the regulatory effect of vasoinhibins on blood-retinal barrier permeability: Diabetic milieu makes a difference. Sci. Rep..

[160] Phuong T.T.T., Redmon S.N., Yarishkin O., Winter J.M., Li D.Y., Križaj D. (2017). Calcium influx through TRPV4 channels modulates the adherens contacts between retinal microvascular endothelial cells. J. Physiol..

[161] Bu S.C., Kuijer R., Li X.R., Hooymans J.M., Los L.I. (2014). Idiopathic epiretinal membrane. Retina.

[162] Pierro L., Zampedri E., Milani P., Gagliardi M., Isola V., Pece A. (2012). Spectral domain OCT versus time domain OCT in the evaluation of macular features related to wet age-related macular degeneration. Clin. Ophthalmol..

[163] Takahashi E., Fukushima A., Haga A., Inomata Y., Ito Y., Fukushima M., Tanihara H. (2016). Effects of mechanical stress and vitreous samples in retinal pigment epithelial cells. Biochem. Biophys. Res. Commun..

[164] Sarks S.H. (1976). Ageing and degeneration in the macular region: A clinico-pathological study. Br. J. Ophthalmol..

[165] Lutty G., Grunwald J., Majji A.B., Uyama M., Yoneya S. (1999). Changes in choriocapillaris and retinal pigment epithelium in age-related macular degeneration. Mol. Vis..

[166] McLeod D.S., Grebe R., Bhutto I., Merges C., Baba T., Lutty G.A. (2009). Relationship between RPE and choriocapillaris in age-related macular degeneration. Investig. Ophthalmol. Vis. Sci..

[167] Rafii S., Butler J.M., Ding B.S. (2016). Angiocrine functions of organ-specific endothelial cells. Nature.

[168] Saint-Geniez M., Kurihara T., Sekiyama E., Maldonado A.E., D’Amore P.A. (2009). An essential role for RPE-derived soluble VEGF in the maintenance of the choriocapillaris. Proc. Natl. Acad. Sci. USA.

[169] Marneros A.G., Fan J., Yokoyama Y., Gerber H.P., Ferrara N., Crouch R.K., Olsen B.R. (2005). Vascular endothelial growth factor expression in the retinal pigment epithelium is essential for choriocapillaris development and visual function. Am. J. Pathol..

[170] Gaudric A., Sterkers M., Coscas G. (1987). Retinal detachment after choroidal ischemia. Am. J. Ophthalmol..

[171] Saito Y., Tano Y. (1998). Retinal pigment epithelial lesions associated with choroidal ischemia in preeclampsia. Retina.

[172] Spencer C., Abend S., McHugh K.J., Saint-Geniez M. (2017). Identification of a synergistic interaction between endothelial cells and retinal pigment epithelium. J. Cell Mol. Med..

[173] Benedicto I., Lehmann G.L., Ginsberg M., Nolan D.J., Bareja R., Elemento O., Salfati Z., Alam N.M., Prusky G.T., Llanos P. (2017). Concerted regulation of retinal pigment epithelium basement membrane and barrier function by angiocrine factors. Nat. Commun..

[174] Ohlmann A., Scholz M., Koch M., Tamm E.R. (2016). Epithelial-mesenchymal transition of the retinal pigment epithelium causes choriocapillaris atrophy. Histochem. Cell Biol..

[175] Suzuki M., Kamei M., Itabe H., Yoneda K., Bando H., Kume N., Tano Y. (2007). Oxidized phospholipids in the macula increase with age and in eyes with age-related macular degeneration. Mol. Vis..

[176] Yamada Y., Tian J., Yang Y., Cutler R.G., Wu T., Telljohann R.S., Mattson M.P., Handa J.T. (2008). Oxidized low density lipoproteins induce a pathologic response by retinal pigmented epithelial cells. J. Neurochem..

[177] Gnanaguru G., Choi A.R., Amarnani D., D’Amore P.A. (2016). Oxidized Lipoprotein Uptake Through the CD36 Receptor Activates the NLRP3 Inflammasome in Human Retinal Pigment Epithelial Cells. Investig. Ophthalmol. Vis. Sci..

[178] Kozlowski M.R. (2012). RPE cell senescence: A key contributor to age-related macular degeneration. Med. Hypotheses.

[179] Wu T., Xu W., Wang Y., Tao M., Hu Z., Lv B., Hui Y., Du H. (2020). OxLDL enhances choroidal neovascularization lesion through inducing vascular endothelium to mesenchymal transition process and angiogenic factor expression. Cell Signal..

[180] Fukushima A., Takahashi E., Saruwatari J., Tanihara H., Inoue T. (2020). The angiogenic effects of exosomes secreted from retinal pigment epithelial cells on endothelial cells. Biochem. Biophys. Rep..

[181] Bastiaans J., van Meurs J.C., van Holten-Neelen C., Nagtzaam N.M., van Hagen P.M., Chambers R.C., Hooijkaas H., Dik W.A. (2013). Thrombin induces epithelial-mesenchymal transition and collagen production by retinal pigment epithelial cells via autocrine PDGF-receptor signaling. Investig. Ophthalmol. Vis. Sci..

[182] McLenachan S., Hao E., Zhang D., Zhang L., Edel M., Chen F. (2017). Bioengineered Bruch’s-like extracellular matrix promotes retinal pigment epithelial differentiation. Biochem. Biophys. Rep..

[183] Friedman E. (2000). The role of the atherosclerotic process in the pathogenesis of age-related macular degeneration. Am. J. Ophthalmol..

[184] Nackman G.B., Karkowski F.J., Halpern V.J., Gaetz H.P., Tilson M.D. (1997). Elastin degradation products induce adventitial angiogenesis in the Anidjar/Dobrin rat aneurysm model. Surgery.

[185] Maminishkis A., Chen S., Jalickee S., Banzon T., Shi G., Wang F.E., Ehalt T., Hammer J.A., Miller S.S. (2006). Confluent monolayers of cultured human fetal retinal pigment epithelium exhibit morphology and physiology of native tissue. Investig. Ophthalmol. Vis. Sci..

[186] Sonoda S., Spee C., Barron E., Ryan S.J., Kannan R., Hinton D.R. (2009). A protocol for the culture and differentiation of highly polarized human retinal pigment epithelial cells. Nat. Protoc..

[187] Toops K.A., Tan L.X., Lakkaraju A. (2014). A detailed three-step protocol for live imaging of intracellular traffic in polarized primary porcine RPE monolayers. Exp. Eye Res..

[188] Das S., Becker B.N., Hoffmann F.M., Mertz J.E. (2009). Complete reversal of epithelial to mesenchymal transition requires inhibition of both ZEB expression and the Rho pathway. BMC Cell Biol..

[189] Furihata T., Kawamatsu S., Ito R., Saito K., Suzuki S., Kishida S., Saito Y., Kamiichi A., Chiba K. (2015). Hydrocortisone enhances the barrier properties of HBMEC/ciβ, a brain microvascular endothelial cell line, through mesenchymal-to-endothelial transition-like effects. Fluids Barriers CNS.

[190] Tezel T.H., Del Priore L.V. (1997). Reattachment to a substrate prevents apoptosis of human retinal pigment epithelium. Graefes Arch. Clin. Exp. Ophthalmol..

[191] Tezel T.H., Del Priore L.V., Kaplan H.J. (2004). Reengineering of aged Bruch’s membrane to enhance retinal pigment epithelium repopulation. Investig. Ophthalmol. Vis. Sci..

[192] Phillips S.J., Sadda S.R., Tso M.O., Humayan M.S., de Juan E., Binder S. (2003). Autologous transplantation of retinal pigment epithelium after mechanical debridement of Bruch’s membrane. Curr. Eye Res..

[193] Del Priore L.V., Geng L., Tezel T.H., Kaplan H.J. (2002). Extracellular matrix ligands promote RPE attachment to inner Bruch’s membrane. Curr. Eye Res..

[194] Chan E.S., Cronstein B.N. (2010). Methotrexate—How does it really work?. Nat. Rev. Rheumatol..

[195] Gangaputra S., Newcomb C.W., Liesegang T.L., Kacmaz R.O., Jabs D.A., Levy-Clarke G.A., Nussenblatt R.B., Rosenbaum J.T., Suhler E.B., Thorne J.E. (2009). Methotrexate for ocular inflammatory diseases. Ophthalmology.

[196] Frenkel S., Hendler K., Siegal T., Shalom E., Pe’er J. (2008). Intravitreal methotrexate for treating vitreoretinal lymphoma: 10 years of experience. Br. J. Ophthalmol..

[197] Sadaka A., Sisk R.A., Osher J.M., Toygar O., Duncan M.K., Riemann C.D. (2016). Intravitreal methotrexate infusion for proliferative vitreoretinopathy. Clin. Ophthalmol..

[198] ClinicalTrials.gov U.S. National Library Medicine The GUARD Trial—Part 1: A Phase 3 Clinical Trial for Prevention of Proliferative Vitreoretinopathy. https://clinicaltrials.gov/ct2/show/NCT04136366.

[199] Kurup S.K., Gee C., Greven C.M. (2010). Intravitreal methotrexate in therapeutically resistant exudative age-related macular degeneration. Acta Ophthalmol..

[200] Lam J.D., Oh D.J., Wong L.L., Amarnani D., Park-Windhol C., Sanchez A.V., Cardona-Velez J., McGuone D., Stemmer-Rachamimov A.O., Eliott D. (2017). Identification of RUNX1 as a Mediator of Aberrant Retinal Angiogenesis. Diabetes.

[201] Espinosa-Heidmann D.G., Reinoso M.A., Pina Y., Csaky K.G., Caicedo A., Cousins S.W. (2005). Quantitative enumeration of vascular smooth muscle cells and endothelial cells derived from bone marrow precursors in experimental choroidal neovascularization. Exp. Eye Res..

[202] Little K., Ma J.H., Yang N., Chen M., Xu H. (2018). Myofibroblasts in macular fibrosis secondary to neovascular age-related macular degeneration—The potential sources and molecular cues for their recruitment and activation. EBioMedicine.

[203] Luo X., Yang S., Liang J., Zhai Y., Shen M., Sun J., Feng Y., Lu X., Zhu H., Wang F. (2018). Choroidal pericytes promote subretinal fibrosis after experimental photocoagulation. Dis. Models Mech..

